# Association of Treatment With Medications for Opioid Use Disorder With Mortality After Hospitalization for Injection Drug Use–Associated Infective Endocarditis

**DOI:** 10.1001/jamanetworkopen.2020.16228

**Published:** 2020-10-14

**Authors:** Simeon D. Kimmel, Alexander Y. Walley, Yijing Li, Benjamin P. Linas, Sara Lodi, Dana Bernson, Roger D. Weiss, Jeffrey H. Samet, Marc R. Larochelle

**Affiliations:** 1 Section of General Internal Medicine, Department of Medicine, Boston Medical Center, Boston, Massachusetts; 2 Section of Infectious Diseases, Department of Medicine, Boston Medical Center, Boston, Massachusetts; 3 Boston University School of Medicine, Boston, Massachusetts; 4 Massachusetts Department of Public Health, Boston, Massachusetts; 5 Department of Biostatistics, Boston University School of Public Health, Boston, Massachusetts; 6 Substance Use Disorders Division, McLean Hospital, Belmont, Massachusetts; 7 Harvard Medical School, Belmont, Massachusetts

## Abstract

**Importance:**

Although hospitalizations for injection drug use–associated infective endocarditis (IDU-IE) have increased during the opioid crisis, utilization of and mortality associated with receipt of medication for opioid use disorder (MOUD) after discharge from the hospital among patients with IDU-IE are unknown.

**Objective:**

To assess the proportion of patients receiving MOUD after hospitalization for IDU-IE and the association of MOUD receipt with mortality.

**Design, Setting, and Participants:**

This retrospective cohort study used a population registry with person-level medical claims, prescription monitoring program, mortality, and substance use treatment data from Massachusetts between January 1, 2011, and December 31, 2015; IDU-IE–related discharges between July 1, 2011, and June, 30, 2015, were analyzed. All Massachusetts residents aged 18 to 64 years with a first hospitalization for IDU-IE were included; IDU-IE was defined as any hospitalization with a diagnosis of endocarditis and at least 1 claim in the prior 6 months for OUD, drug use, or hepatitis C and with 2-month survival after hospital discharge. Data were analyzed from November 11, 2018, to June 23, 2020.

**Exposure:**

Receipt of MOUD, defined as any treatment with methadone, buprenorphine, or naltrexone, within 3 months after hospital discharge excluding discharge month for IDU-IE.

**Main Outcomes and Measures:**

The main outcome was all-cause mortality. The proportion of patients who received MOUD in the 3 months after hospital discharge was calculated. Multivariable Cox proportional hazard regression models were used to examine the association of MOUD receipt with mortality, adjusting for sex, age, medical and psychiatric comorbidities, and homelessness. In the secondary analysis, receipt of MOUD was considered as a monthly time-varying exposure.

**Results:**

Of 679 individuals with IDU-IE, 413 (60.8%) were male, the mean (SD) age was 39.2 (12.1) years, 298 (43.9%) were aged 18 to 34 years, 419 (72.3) had mental illness, and 209 (30.8) experienced homelessness. A total of 134 individuals (19.7%) received MOUD in the 3 months before hospitalization and 165 (24.3%) in the 3 months after hospital discharge. Of those who received MOUD after discharge, 112 (67.9%) received buprenorphine. The crude mortality rate was 9.2 deaths per 100 person-years. MOUD receipt within 3 months after discharge was not associated with reduced mortality (adjusted hazard ratio, 1.29; 95% CI, 0.61-2.72); however, MOUD receipt was associated with reduced mortality in the month that MOUD was received (adjusted hazard ratio, 0.30; 95% CI, 0.10-0.89).

**Conclusions and Relevance:**

In this cohort study, receipt of MOUD was associated with reduced mortality after hospitalization for injection drug use–associated endocarditis only in the month it was received. Efforts to improve MOUD initiation and retention after IDU-IE hospitalization may be beneficial.

## Key Points

**Question:**

Is there an association between receipt of medication for opioid use disorder (MOUD) and mortality after hospitalization for injection drug use–associated infective endocarditis?

**Findings:**

In this cohort study 679 individuals hospitalized with injection drug use–associated endocarditis, 24% received MOUD within 3 months of discharge. MOUD receipt within 3 months of discharge was not associated with reduced mortality but was associated with a reduction in mortality in the month received.

**Meaning:**

In this study, treatment with MOUD was uncommon and was associated with reduced mortality in the time-varying analysis but not the main analysis, possibly owing to poor treatment retention.

## Teaser

This cohort study assess the association of receipt of medication for opioid use disorder and mortality after hospitalization for injection drug use–associated infective endocarditis in Massachusetts.

## Introduction

During the opioid crisis in the US, hospitalizations for injection drug use–associated infective endocarditis (IDU-IE) doubled between 2008 and 2014 and now account for 10% of approximately 40 000 annual hospitalizations for IE.^1^ Treatment for IDU-IE includes weeks of intravenous antibiotics and sometimes valve surgery,^2^ but mortality remains between 5% to 8% in-hospital and 16% to 34% 1 year after discharge.^3,4,5^ Although hospitalizations for IDU-IE are opportunities to initiate medication for opioid use disorder (MOUD), including methadone, buprenorphine, and extended release naltrexone,^6^ several studies demonstrate they are rarely offered.^7,8,9^

MOUD has been extensively investigated,^10,11,12,13^ but data describing the association of MOUD receipt with mortality after IDU-IE–related hospitalization are limited.^3,4,14,15^ A national study^9^ of privately insured individuals with IDU-IE showed that MOUD was associated with decreased rehospitalizations, but smaller studies^3,4,14,15^ investigating mortality have had conflicting results. Studies in Ontario^4^ and Boston^14^ found no association between MOUD receipt and long-term mortality in patients with IDU-IE; a small study in Maine^15^ showed that MOUD was associated with decreased 90-day mortality only in unadjusted analyses.^3^ These studies were limited by small samples or incomplete assessment of MOUD receipt.

With an OUD prevalence of approximately 5%, Massachusetts has the second-highest rate of opioid-related inpatient hospitalizations and double the national average for deaths from opioid overdose (28.2 deaths per 100 000 persons in 2017).^16,17,18^ To investigate this crisis, the Massachusetts legislature passed the Chapter 55 Acts of 2015, which permitted individual-level data linkage from multiple Massachusetts government agencies, now called the Massachusetts Public Health Data Warehouse (PHD).^18^ We used the PHD to identify individuals hospitalized for IDU-IE and to characterize treatment with MOUD before and after the hospitalization. We evaluated whether receipt of MOUD after discharge for IDU-IE was associated with reduced all-cause mortality in the following year.

## Methods

### Study Design and Data Source

We performed a retrospective cohort study using the PHD, which includes data from January 1, 2011, to December 31, 2015. Residents of Massachusetts aged 11 or older with an insurance claim in the All-Payer Claims Database are included (approximately 98% of residents). Records from the All-Payer Claims Database link longitudinally at the person level to 16 data sets with administrative records from government agencies using a multistage deterministic linkage technique that has been previously described.^18^ The PHD was established by Massachusetts law and has been used to study MOUD receipt after overdose and during the perinatal period and to estimate the statewide OUD prevalence.^11,19,20^ For this study, we used data from the All-Payer Claims Database, the Registry of Vital Records and Statistics, the Prescription Monitoring Program, the Acute Care Hospital Case Mix, the Bureau of Substance Addiction Services licensed treatment encounters, the Department of Mental Health, and the Massachusetts Ambulance Trip Record Information System. In the PHD, counts smaller than 10 are suppressed to protect privacy. The Boston University Medical Campus Institutional Review Board determined this study was not human subjects research. This study followed the Strengthening the Reporting of Observational Studies in Epidemiology (STROBE) reporting guideline.^21^

### Cohort Selection

The cohort includes individuals aged 18 to 64 years at the time of endocarditis diagnosis. We excluded individuals older than 64 years to prevent misclassification of those with endocarditis more likely attributable to noninjection risk factors (eg, dialysis or cardiac procedures). We excluded those younger than 18 years because they are less likely to be treated with MOUD.^22,23^

We identified individuals with a discharge from an acute care hospital with a diagnosis of endocarditis in the PHD using *International Classification of Diseases, Ninth Revision (ICD-9)* codes (421.0, 421.9, 424.90, 424.91, and 424.99) between July 1, 2011, and June 30, 2015 to allow for at least 6 months of observation before and after the hospitalization (eFigure 1 in the Supplement). Next, we identified endocarditis cases associated with injection drug use based on evidence of 1 or more of the following in the 6 months before hospital discharge: previous medical claims with ICD-9 diagnosis codes of drug use or drug treatment for opioids, cocaine, or amphetamines; OUD; or hepatitis C virus infection (eFigure 1 and eTable 1 in the Supplement); admission to a Bureau of Substance Addiction Services addiction treatment service for OUD; or receipt of MOUD. We adapted a validated algorithm that used *International Statistical Classification of Diseases and Related Health Problems, Tenth Revision* inpatient diagnosis codes for endocarditis with hepatitis C or drug use disorders (opioids, cocaine, stimulants, and psychoactive substances) that had a sensitivity of 93%, specificity of 61%, and positive predictive value of 83% (eTable 1 in the Supplement).^24^

We included only the first hospital discharge (defined as the index discharge) for individuals with multiple qualifying endocarditis-related hospital discharges. In addition, because many individuals received intravenous antibiotics for up to 6 weeks in settings where we were unable to observe admission dates or MOUD receipt (skilled nursing facility or long-term acute care hospitals), we restricted the analysis to individuals who survived at least 2 months after the index discharge.

### Exposure and Outcome

We identified exposure to MOUD in monthly intervals after IDU-IE–related hospital discharge. We determined methadone receipt through a medical claim for methadone administration via Healthcare Common Procedural Coding System code H0020 or record of treatment from the Bureau of Substance Addiction Services. We used the Prescription Monitoring Program to ascertain dispensed buprenorphine or buprenorphine-naloxone. Naltrexone receipt was determined from a pharmacy claim for injectable or oral naltrexone. Inpatient MOUD administration is not included in the PHD.

We used 3 definitions of MOUD exposures. In our primary analysis, we classified individuals’ MOUD status once after discharge as having received MOUD if it was received in the 3-month period after the IDU-IE–related hospital discharge; we excluded the month of discharge because the medication could have been received before the hospitalization. This approach is similar to an intent-to-treat analysis. Because individuals with IDU-IE often receive post–acute medical care when MOUD receipt cannot be observed, we only included individuals who survived at least 2 months after discharge to ensure an opportunity to classify MOUD exposure (eFigures 1 and 2 in the Supplement).

We used 2 additional as-treated approaches defining MOUD receipt as time-varying exposures as described previously^11^ (eFigures 1 and 2 in the Supplement). For an on-treatment classification, we considered individuals exposed to MOUD in any month in which it was received. For a through-discontinuation classification, we defined MOUD exposure as any month when MOUD was received and the month following last receipt. The through-discontinuation approach attributes the increased risk associated with MOUD discontinuation to MOUD. The primary outcome was all-cause mortality in the PHD up to 1 year after discharge.

### Confounders

Potential confounders included in multivariable models were sex, age, mental illness, medical comorbidities determined by the Elixhauser Comorbidity Index, homelessness, and MOUD receipt in the 3 months before the hospital admission for IDU-IE. We examined records for each individual in the preceding 6 months, including the index IDU-IE–related hospital discharge. However, certain variables were only designated as present or absent at any time in the 5-year time frame of the PHD. We used the PHD to determine sex and age at hospital discharge, with age categorized as 18 to 34, 35 to 49, and 50 to 64 years. We identified mental illness, defined as anxiety, depression, or psychosis (eTable 2 in the Supplement). We calculated an Elixhauser Comorbidity Index using the presence of 31 comorbidities identified by *ICD-9* diagnosis codes to estimate illness severity (eTable 3 in the Supplement).^25^ The *ICD-9* codes for mental illness and the Elixhauser Comorbidity Index were identified at any point in the PHD. We identified homelessness with 1 of the following variables at least once in the PHD: a diagnosis code for homelessness from the All Payer Claims Database (*ICD-9* V60), Department of Mental Health Data recording loss of housing, an ambulance encounter in which the word *homeless* or *shelter* appeared in the narrative report, or a controlled substance prescription record with a shelter as the patient’s address. We identified MOUD receipt in the 3 months before the hospitalization for IDU-IE using the same methods as for the exposure. In addition, we determined receipt of prescription opioid analgesic medications (excluding buprenorphine) before and after the IDU-IE–related hospitalization from the Prescription Monitoring Program. Receipt of prescription opioid analgesic medications was excluded from the models owing to limited degrees of freedom.

### Statistical Analyses

Data were analyzed from November 11, 2018, to June 23, 2020. We used χ^2^ tests to compare baseline characteristics by receipt of MOUD. We examined time to all-cause mortality by receipt of MOUD using Cox proportional hazards regression models adjusting for potential confounders using the 3 aforementioned MOUD exposure definitions. For model 1 (intention to treat), we used a Kaplan-Meier test to estimate the cumulative incidence of all-cause mortality. For model 2 (monthly MOUD receipt) and model 3 (monthly MOUD receipt through discontinuation), we used an extended Kaplan-Meier test to estimate the cumulative incidence of all-cause mortality as time-varying exposures. Two-sided *P* < .05 was considered statistically significant. Analyses were performed using SAS Studio, version 3.6 (SAS Institute).

We performed sensitivity analyses in which the upper age limit and then hepatitis C were removed from the inclusion criteria. We repeated analyses that included only individuals who survived through the discharge month.

## Results

### Baseline Characteristics

We identified 2706 endocarditis-related hospitalizations, 2294 (84.8%) of which included 6 months of observations before and after discharge; 1231 (45.5%) were classified as hospitalizations for IDU-IE. Among these IDU-IE–related hospitalizations, 755 (61.3%) were the first hospitalization for IDU-IE; in 703 hospitalizations (57.1%), the individual survived until discharge, and in 679 hospitalizations (55.2%), the individual survived 2 months after discharge (eFigure 3 in the Supplement).

Of the 679 individuals with IDU-IE–related hospital discharges, 413 (60.8%) were male; the mean (SD) age was 39.2 (12.1) years, 298 (43.9%) were aged 18 to 34 years; 491 (72.3%) experienced high rates of anxiety, depression, or psychosis; and 209 (30.8%) experienced homelessness. A total of 196 individuals (28.9%) received a prescription for an opioid analgesic in the 6 months before the IDU-IE–related hospitalization, and 225 (33.1%) received a prescription in the 3 months after. Of those with an opioid analgesic prescription before the hospitalization, 35 (17.9%) received MOUD. Characteristics associated with receipt of MOUD after IDU-IE included previous receipt of MOUD, young age, and female sex. Receipt of an opioid analgesic prescription in the 6 months before IDU-IE was negatively associated with MOUD receipt. There were no differences in Elixhauser Comorbidity Index scores or the proportion with homelessness among those who did or did not receive MOUD (Table 1).

**Table 1.** Characteristics of Individuals With IDU-IE by Receipt of MOUD After Hospital Discharge From July 2011 to June 2015

Characteristics	Individuals, No. (%)			*P* value^a^
	Total (N = 679)	MOUD within 3 mo after hospitalization for IDU-IE		
		No (n = 514)	Yes (n = 165)	
MOUD 6 mo before admission	173 (25.5)	70 (13.6)	103 (62.4)	<.001
MOUD 3 mo before admission,	134 (19.7)	48 (9.3)	86 (52.1)	<.001
Age, y				.002
18-34	298 (43.9)	207 (40.3)	91(55.2)	NA
35-49	214 (31.5)	168 (32.7)	46 (27.9)	NA
50-64	167 (24.6)	139 (27.0)	28 (17.0)	NA
Male	413 (60.8)	323 (62.8)	90 (54.6)	.06
Mental illness^b^	491 (72.3)	358 (69.7)	133 (80.6)	.006
Homelessness^c^	209 (30.8)	152 (29.6)	57 (34.6)	.23
Elixhauser Comorbidity Index, median (IQR)^d^	8 [5-12]	8 [4-12]	8 [5-10]	.26
Opioid prescribed 6 mo before admission	196 (28.9)	161 (31.3)	35 (21.2)	.01

Abbreviations: IDU-IE, injection drug use–associated infective endocarditis; MOUD, medication for opioid use disorder; NA, not applicable.

^a^ *P* values represent results from χ^2^ tests comparing demographic characteristics between those who did and did not receive MOUD within 3 months after IDU-IE–related discharge.

^b^ Mental illness was defined as anxiety, depression, or psychosis during any point in the study period.

^c^ Homelessness was defined based on the Master Demographic file of the Massachusetts Public Health Data Set, which included the diagnosis code for homelessness from All-Payer Claims Database or CaseMix; the Department of Mental Health Data set recording loss of housing; an ambulance encounter in which the word *homeless* or *shelter* appeared in the narrative report; or a controlled substance prescription in which the patient’s address was a shelter.

^d^ The Elixhauser Comorbidity Index was calculated using 31 comorbidities identified from All-Payer Claims Database codes.^25^

### Receipt of MOUD Before and After Hospitalization for IDU-IE

**Figure 1.**
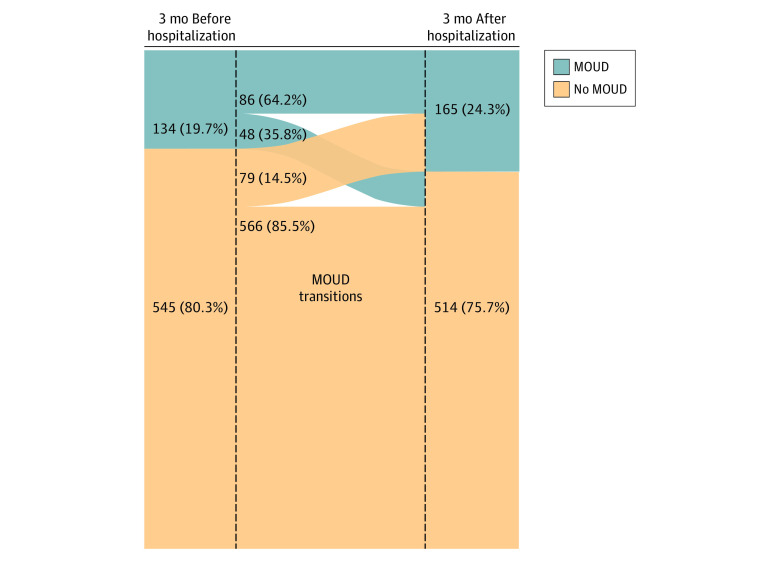
Receipt of Medication for Opioid Use Disorder (MOUD) Before and After Hospitalization for Injection Drug Use–Associated Infective Endocarditis From July 2011 to June 2015 Receipt of MOUD did not include the month of hospitalization.

**Figure 2.**
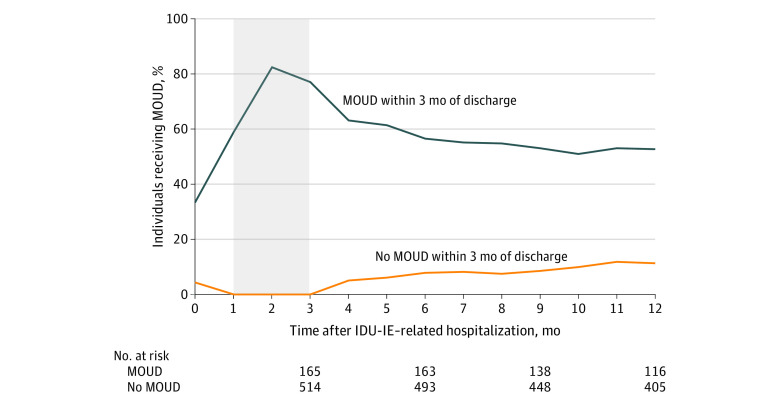
Monthly Receipt of Medication for Opioid Use Disorder (MOUD) by Those Who Did and Did Not Receive MOUD Within 3 Months of Hospital Discharge for Injection Drug Use–Associated Infective Endocarditis (IDU-IE) From July 2011 to June 2015 Primary MOUD exposure was defined as MOUD receipt in months 1 through 3 (shaded area). Month 0 was excluded because MOUD could have been received before hospitalization.

Of the individuals with IDU-IE, 134 (19.7%) received MOUD in the 3 months before the IDU-IE–related hospitalization. In the 3 months after the IDU-IE–related hospitalization, the proportion who received MOUD increased 4.6% to 24.3% (165 individuals). Individuals with IDU-IE were more likely to stop existing MOUD treatment than they were to start new MOUD after hospitalization. Of the individuals who had received MOUD in the 3 months before the IDU-IE–related hospitalization, 48 (35.8%) stopped in the 3 months after the hospitalization. In contrast, 79 individuals (14.5%) who did not receive MOUD before hospitalization received MOUD in the 3 months after the hospitalization (Figure 1). Of those who received MOUD, buprenorphine was the most common treatment before (86 [64.2%]) and after (112 [67.9%]) hospitalization. Breakdowns among methadone, extended-release naltrexone, and more than 1 therapy were suppressed to protect privacy owing to small counts. Among those remaining in the cohort 12 months after hospital discharge, MOUD was received by 61 of 116 (52.6%) who received MOUD in the 3 months after discharge and by 46 of 405 (11.4%) who did not receive MOUD in 3 months after discharge (Figure 2).

### All-Cause Mortality

In this cohort of 679 individuals who survived 2 months after the index IDU-IE–related hospital discharge, 61 (8.9%) died in the following 10 months. The crude mortality rate was 9.2 deaths per 100 person-years. Ten or fewer of these deaths were attributable to opioid-related overdose (data were suppressed owing to small counts). Among those excluded owing to death in the first 2 months after discharge, none of the deaths were attributed to opioid overdose.

### Primary, Secondary, and Sensitivity Analyses

**Figure 3.**
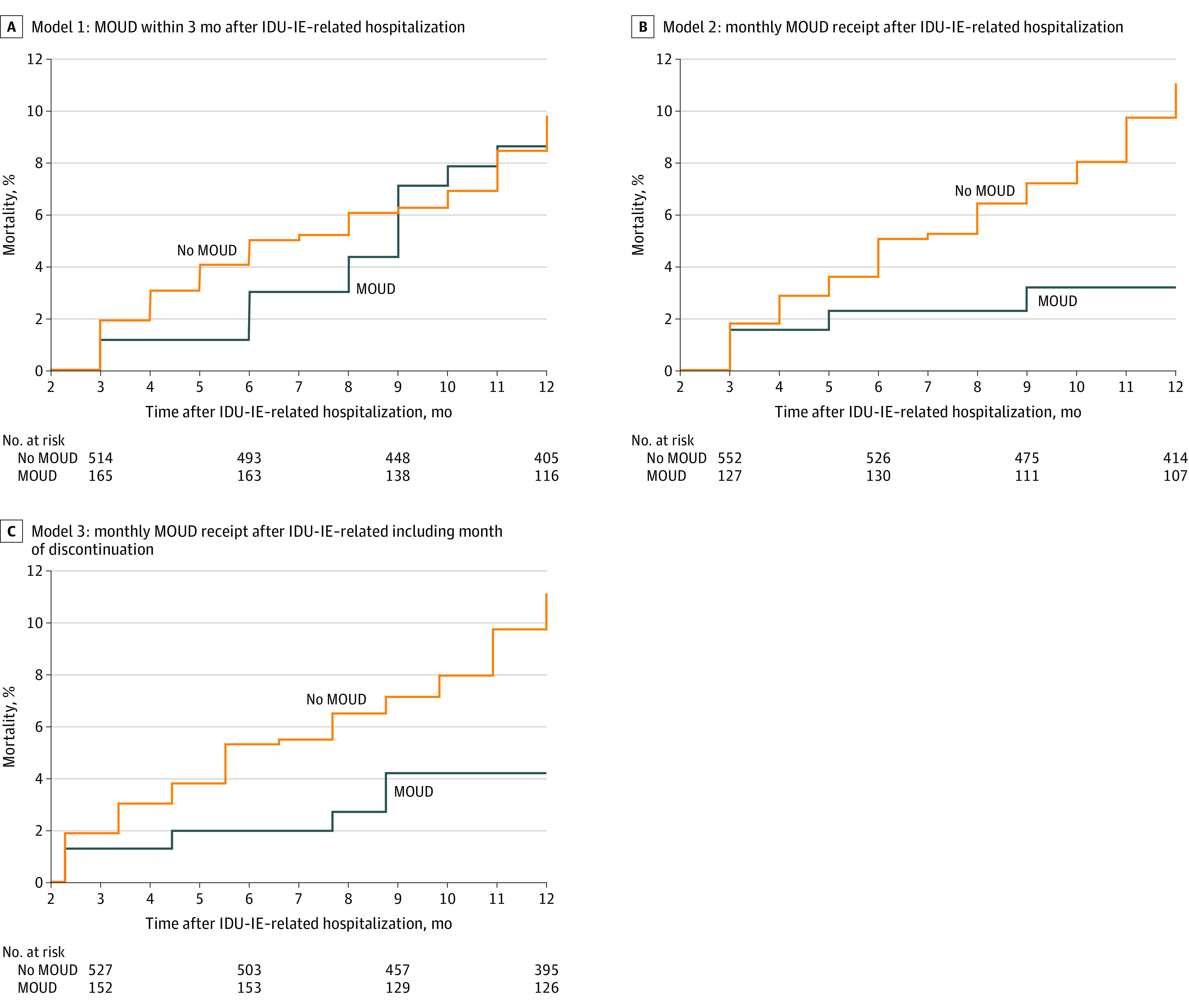
Kaplan-Meier and Extended Kaplan-Meier Cumulative Incidence of All-Cause Mortality by Monthly Exposure to Medication for Opioid Use Disorder (MOUD) for Injection Drug Use–Associated Infective Endocarditis (IDU-IE) From July 2011 to June 2015 Models 1 to 3 included only individuals who survived 2 months after hospitalization for IDU-IE.

In the primary analysis (intent-to-treat approach) in which the exposure classification was determined once and fixed, any receipt of MOUD within 3 months of discharge was not associated with all-cause mortality (adjusted hazard ratio [AHR], 1.29; 95% CI, 0.61-2.72). Younger age (18-34 years) compared with older age (50-64 years) was associated with reduced mortality (AHR, 0.24; 95% CI, 0.11-0.51] (Table 2 and Figure 3).

**Table 2.** Multivariable Cox Proportional Hazards Regression Models for All-Cause Mortality Using Intent-to-Treat and As-Treated Approaches to MOUD Exposure for IDU-IE–Associated Discharges From July 2011 to June 2015^a^

Characteristic	Hazard Ratio (95% CI)		
	Model 1: MOUD within 3 mo after IDU-IE	Model 2: monthly MOUD receipt after IDU-IE	Model 3: monthly after IDU-IE, including month of discontinuation
MOUD	1.29 (0.61-2.72)	0.30 (0.10-0.89)	0.39 (0.15-1.00)
MOUD 3 mo before admission	0.77 (0.34-1.75)	1.35 (0.65-2.81)	1.33 (0.62-2.82)
Age, y			
18-34	0.24 (0.11-0.51)	0.26 (0.12-0.55)	0.26 (0.12-0.55)
35-49	0.60 (0.34-1.06)	0.62 (0.35-1.10)	0.62 (0.35-1.1)
50-64	1 [Reference]	1 [Reference]	1 [Reference]
Female	0.80 (0.45-1.42)	0.80 (0.45-1.43)	0.80 (0.42-1.58)
Elixhauser Comorbidity Index	1.03 (0.97-1.09)	1.03 (0.97-1.09)	1.01 (0.96-1.08)
Mental illness	0.77 (0.39-1.49)	0.81 (0.42-1.57)	0.81 (0.42-1.58)
Homelessness	0.60 (0.32-1.6)	0.60 (0.31-1.14)	0.61 (0.32-1.2)

Abbreviations: IDU-IE, injection drug use–associated infective endocarditis; MOUD, medication for opioid use disorder.

^a^ Homelessness, major mental illness, and Elixhauser Comorbidity Index covariates were defined as evidence during any part of the study period not only before the index IDU-IE–related hospitalization. There were 61 deaths in each model.

With use of the time-varying on-treatment exposure classification, MOUD receipt was associated with 70% decreased mortality (AHR, 0.30; 95% CI, 0.10-0.89) after the IDU-IE–related hospitalization. With use of the time-varying through-discontinuation exposure classification, MOUD was similarly associated with reduced mortality (AHR, 0.39; 95% CI, 0.15-1.00) (Table 2 and Figure 3). In the sensitivity analysis, the results were similar when hepatitis C or age older than 64 years was not considered as an inclusion criterion or when including only individuals who survived through the discharge month (eTable 4 in the Supplement).

## Discussion

In this cohort of 679 individuals with IDU-IE who survived 2 months after hospital discharge, mortality in the following 10 months was 9.2 per 100 person-years. For comparison, mortality in the year following opioid overdose, a risk factor for fatal overdose, was 4.7 deaths per 100 person-years in Massachusetts.^11^ We found that MOUD receipt in the 3 months after hospitalization alone was not associated with reduced long term mortality (model 1). However, we observed a 70% reduction in mortality in the month that MOUD was received (model 2). No mortality benefit was likely observed in the intention-to-treat model (model 1) because more than 40% of individuals in the MOUD group stopped treatment within 6 months of discharge. This suggests that MOUD initiation after IDU-IE alone is insufficient to improve mortality. Efforts to improve retention with MOUD treatment are also necessary and may lead to reduced mortality after IDU-IE. Young age was associated with decreased mortality, but homelessness and mental illness were not associated, perhaps owing to increased clinical engagement.

Less than a quarter of individuals received MOUD in the 3 months after IDU-IE–related hospital discharge, which was 4.6% more than those who received MOUD before hospitalization. Furthermore, only 14.5% of individuals without prior MOUD treatment initiated MOUD after the IDU-IE–related hospitalization. Individuals who previously received opioid analgesic prescriptions (excluding buprenorphine) were also less likely to start MOUD. Overall, individuals were more likely to stop treatment with MOUD after discharge than they were to start treatment. They were more likely to receive opioid analgesia than MOUD. These findings may reflect inpatient providers’ lack of comfort managing MOUD in the context of pain and barriers to continuing MOUD after discharge.^26^

The proportion of individuals with IDU-IE who received MOUD in our study was higher than previously reported treatment rates. Only 16.8% of individuals with IDU-IE received MOUD in Ontario between 2007 and 2016,^4^ and 7.8% had a plan for MOUD at discharge between 2004 and 2014 at a hospital in Boston.^27^ Our study furthers understanding of MOUD treatment after IDU-IE by including monthly treatment receipt after hospital discharge. MOUD has been shown to be associated with reduced rehospitalization but not with mortality after IDU-IE in adjusted analyses.^3,9,11,15^ Previous evidence only showed that referral to outpatient addiction treatment was associated with reduced mortality.^4^ Therefore, to our knowledge, our finding that mortality was reduced among those actively receiving MOUD after IDU-IE is novel. Of note, more than 80% of the deaths in our study were attributed to causes other than opioid overdose. The finding that mortality was reduced in association with monthly MOUD treatment suggests protective effects beyond reduction in overdose. Further studies should explore whether active MOUD treatment is associated with improved antibiotic adherence, increased cardiac surgery, reduced reinfection, or reduced mortality from other causes.

### Strengths and Limitations

This study has strengths. We described all reported IDU-IE–related hospital discharges in Massachusetts. We included outcomes data and granular MOUD receipt. In addition, we analyzed MOUD exposure using multiple approaches.

This study also has limitations. First, we included only individuals who survived 2 months after acute hospital discharge to ensure reliable assignment of MOUD exposure because we were unable to observe MOUD receipt during acute or post–acute hospitalization in skilled nursing or long-term acute care hospitals when MOUD was dispensed from inpatient formularies. We were therefore unable to observe the association of MOUD with mortality during a period with many deaths from endocarditis. However, a sensitivity analysis that included individuals who survived a month or more after discharge produced similar results. Second, discharge dates from post–acute care facilities were not included in the data. Therefore, we chose a 3-month period for the primary MOUD exposure to ensure an opportunity to observe outpatient MOUD receipt. The time-varying monthly MOUD exposure (model 2) was subject to misclassification if individuals were hospitalized for the entire month of observation. We therefore included model 3, an as-treated analysis including the month after discontinuation as part of the exposure, to conservatively attribute MOUD exposure and estimate MOUD effect.^11^ Fourth, this is an observational study subject to confounding by indication. Individuals treated with MOUD may be more likely to complete antibiotics or engage in other treatments. Fifth, we expect some misclassification despite use of a validated algorithm to establish the cohort. There were no substantial differences in the findings when either hepatitis C or the upper age limit was removed from the inclusion criteria. Sixth, owing to data limitations, we were unable to provide clinical details, including the valve involved, whether cardiac surgery was performed, or endocarditis severity. Seventh, also owing to data limitations, major mental illness, Elixhauser Comorbidity Index, and homelessness covariates were drawn from All-Payer Claims Database codes found anytime during the study period. Eighth, because this analysis was based on data from Massachusetts between 2011 and 2015, generalizability may be limited. Further examination is needed to determine the effect of MOUD in years after this study when multiple Massachusetts hospitals improved MOUD delivery.^8,28,29^

## Conclusions

In this cohort study, receipt of MOUD in the 3 months after the IDU-IE–related hospitalization alone was not associated with improved long term mortality. Receipt of MOUD after the IDU-IE–related hospitalization was associated with reduced mortality only if individuals continued to receive treatment. In addition, we found that hospitalization for IDU-IE was an underused opportunity to initiate MOUD. The findings suggest that patient navigation through MOUD initiation, linkage to an outpatient provider to continue pharmacotherapy, and development, evaluation, and implementation of interventions to improve retention are needed.

## Notes

Supplement. **eFigure 1.** Cohort Construction with Primary Exposure Definition and Follow Up, 2011-2015 **eTable 1.** ICD-9 Codes Used to Define Injection Drug Use **eFigure 2.** Primary, Secondary, and Tertiary MOUD Exposure Classification and 10 Months Follow Up After Index IDU-IE Hospitalization **eTable 2.** ICD-9 Codes Used to Define Mental Illness **eTable 3.** ICD-9 Codes Used to Calculate Elixhauser Scores **eFigure 3.** Cohort Construction, 2011-2015 **eTable 4.** Sensitivity Analyses: Adjusted Cox Proportional Hazard Models for Association of MOUD With All-Cause Mortality, July 2011 to June 2015
