# Supplementary material for: Association of Treatment With Medications for Opioid Use Disorder With Mortality After Hospitalization for Injection Drug Use–Associated Infective Endocarditis
Source: JAMA Netw Open. 2020 Oct 14;3(10):e2016228. doi: 10.1001/jamanetworkopen.2020.16228 (PMC7557514; doi:10.1001/jamanetworkopen.2020.16228)
Supplement: Supplement. — eFigure 1. Cohort Construction with Primary Exposure Definition and Follow Up, 2011-2015 eTable 1. ICD-9 Codes Used to Define Injection Drug Use eFigure 2. Primary, Secondary, and Tertiary MOUD Exposure Classification and 10 Months Follow Up After Index IDU-IE Hospitalization eTable 2. ICD-9 Codes Used to Define Mental Illness eTable 3. ICD-9 Codes Used to Calculate Elixhauser Scores eFigure 3. Cohort Construction, 2011-2015 eTable 4. Sensitivity Analyses: Adjusted Cox Proportional Hazard Models for Association of MOUD With All-Cause Mortality, July 2011 to June 2015 [file jamanetwopen-e2016228-s001.pdf]

Supplemental Online Content

Kimmel SD, Walley AY, Li Y, et al. Association of treatment with medications for opioid use disorder with mortality after hospitalization for injection drug use–associated infective endocarditis. *JAMA Netw Open*. 2020;3(10):e2016228. doi:10.1001/jamanetworkopen.2020.16228

eFigure 1. Cohort Construction with Primary Exposure Definition and Follow Up, 2011-2015

eTable 1. ICD-9 Codes Used to Define Injection Drug Use

eFigure 2. Primary, Secondary, and Tertiary MOUD Exposure Classification and 10 Months Follow Up After Index IDU-IE Hospitalization

eTable 2. ICD-9 Codes Used to Define Mental Illness

eTable 3. ICD-9 Codes Used to Calculate Elixhauser Scores

eFigure 3. Cohort Construction, 2011-2015

eTable 4. Sensitivity Analyses: Adjusted Cox Proportional Hazard Models for Association of MOUD With All-Cause Mortality, July 2011 to June 2015

This supplemental material has been provided by the authors to give readers additional information about their work.

© 2020 Kimmel SD et al. *JAMA Network Open.*

e Figure 1: Cohort Construction with Primary Exposure Definition and Follow Up, 2011-2015

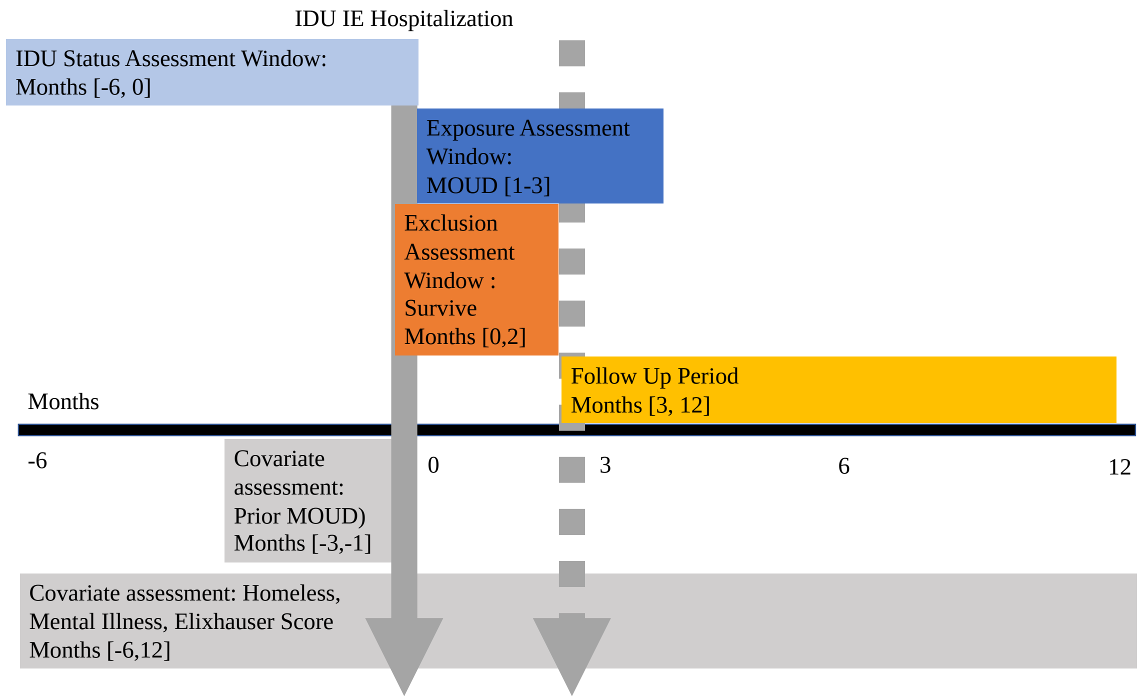

© 2020 Kimmel SD et al. *JAMA Network Open.*

e Table 1: ICD-9 Codes used to Define Injection Drug Use

HCV	ICD9	ICD9 Description
	07041	Acute hepatitis C with hepatic coma
	07044	Chronic hepatitis C with hepatic coma
	07054	Chronic hepatitis C without mention of hepatic coma
	07051	Acute hepatitis C without mention of hepatic coma
	07070	Unspecified viral hepatitis C without hepatic coma
	07071	Unspecified viral hepatitis C with hepatic coma
	V0262	Hepatitis C carrier

OUD	ICD9	ICD9 Description
	30400	Opioid type dependence, unspecified
	30401	Opioid type dependence, continuous
	30402	Opioid type dependence, episodic
	30403	Opioid type dependence, in remission
	30470	Combinations of opioid type drug with any other drug dependence, unspecified
	30471	Combinations of opioid type drug with any other drug dependence, continuous
	30472	Combinations of opioid type drug with any other drug dependence, episodic
	30473	Combinations of opioid type drug with any other drug dependence, in remission
	30550	Opioid abuse, unspecified
	30551	Opioid abuse, continuous
	30552	Opioid abuse, episodic
	30553	Opioid abuse, in remission

Drug Abuse Treatment	ICD9	ICD9 Description
	9463	Alcohol rehabilitation and detoxification
	9462	Alcohol detoxification
	9464	Drug rehabilitation
	9465	Drug detoxification
	9466	Drug rehabilitation and detoxification
	9467	Combined alcohol and drug rehabilitation
	9468	Combined alcohol and drug detoxification
	9469	Combined alcohol and drug rehabilitation and detoxification
	9445	Drug addiction counseling

© 2020 Kimmel SD et al. *JAMA Network Open.*

	9446	Alcoholism counseling

Drug Use	ICD9	ICD9 Description
	E8500	Accidental poisoning by heroin
	E0501	Accidental poisoning by methadone
	E8502	Accidental poisoning by other opiates and related narcotics
	30400	Opioid type dependence, unspecified
	30401	Opioid type dependence, continuous
	30402	Opioid type dependence, episodic
	30403	Opioid type dependence, in remission
	30470	Combinations of opioid type drug with any other drug dependence, unspecified
	30471	Combinations of opioid type drug with any other drug dependence, continuous
	30472	Combinations of opioid type drug with any other drug dependence, episodic
	30473	Combinations of opioid type drug with any other drug dependence, in remission
	30550	Opioid abuse, unspecified
	30551	Opioid abuse, continuous
	30552	Opioid abuse, episodic
	30571	Opioid/other dependence (unspecified)
	30572	Opioid/other dependence (continuous)
	30573	Opioid/other dependence (episodic)
	96500	Poisoning by opium (alkaloids), unspecified
	96501	Poisoning by heroin
	95602	Poisoning by Methadone
	30421	Cocaine dependence, continuous
	30422	Cocaine dependence, episodic
	30423	Cocaine dependence, in remission
	30560	Cocaine abuse, unspecified
	30561	Cocaine abuse, continuous

© 2020 Kimmel SD et al. *JAMA Network Open.*

	30562	Cocaine abuse, episodic
	30563	Cocaine abuse, in remission
	30440	Amphetamine dependence (unspecified)
	30441	Amphetamine and other psychostimulant dependence, continuous
	30442	Amphetamine and other psychostimulant dependence, episodic
	30571	Opioid/other dependence (unspecified)
	30572	Opioid/other dependence (continuous)
	30573	Opioid/other dependence (episodic)
	96970	Poisoning by psychostimulant, unspecified
	96971	Poisoning by caffeine
	96972	Poisoning by amphetamines
	96973	Poisoning by methylphenidate
	96979	Poisoning by other psychostimulants
	2920	Drug withdrawal syndrome- Drug: abstinence syndrome or symptoms, withdrawal syndrome or symptoms
	64833	Drug dependence of mother, antepartum condition or complication
	E8542	Accidental Death Caused by psychostimulants

© 2020 Kimmel SD et al. *JAMA Network Open.*

e Figure 2: Primary,[1] Secondary,[2] and Tertiary[3] MOUD Exposure Classification and 10 Months Follow Up after Index IDU-IE Hospitalization

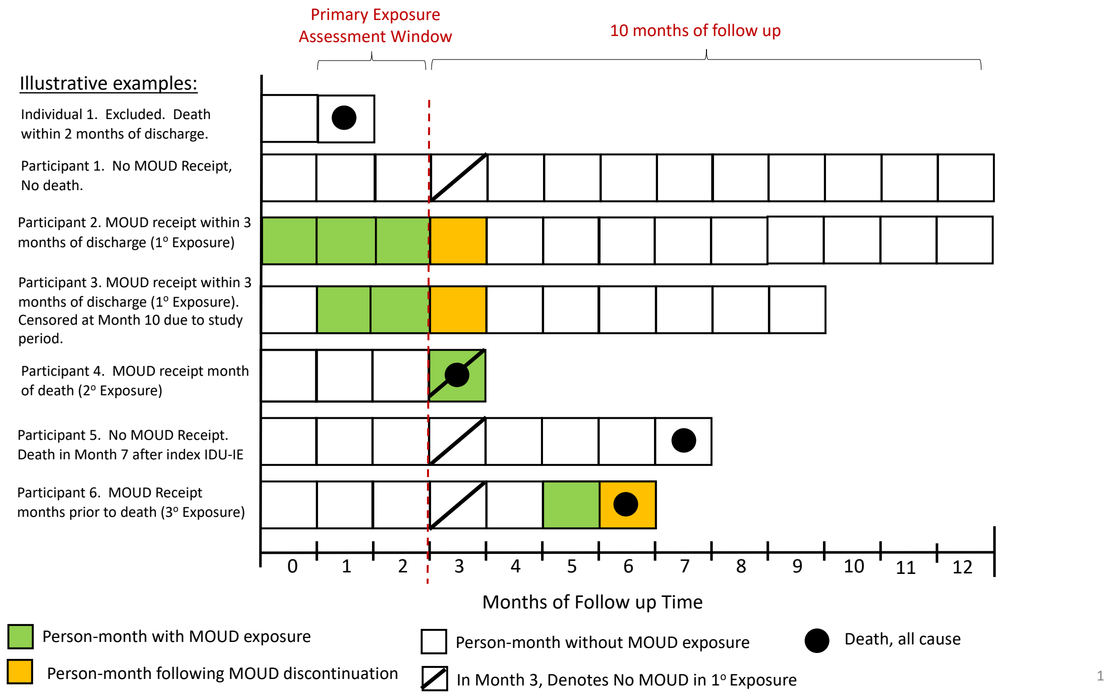

[1]An intent-to-treat approach where individuals are classified as exposed to MOUD if they received any MOUD (buprenorphine, methadone, or naltrexone) in the two months following IDU-IE discharge
[2]An as-treated approach to identify time-varying exposure to MOUD where individuals are considered exposed to MOUD in any month in which it was received
[3]An as-treated approach to identify time-varying exposure to MOUD where individuals are considered exposed to MOUD in any month in which it was received including the month following discontinuation

© 2020 Kimmel SD et al. *JAMA Network Open.*

e Table 2: ICD-9 Codes used to Define Mental Illness

Depression	ICD9	ICD9 Description
	29699	Other specified episodic mood disorder
	29636	Major depressive affective disorder, recurrent episode, in full remission
	29635	Major depressive affective disorder, recurrent episode, in partial or unspecified remission
	29631	Major depressive affective disorder, recurrent episode, mild
	29632	Major depressive affective disorder, recurrent episode, moderate
	29633	Major depressive affective disorder, recurrent episode, severe, without mention of psychotic behavior
	29630	Major depressive affective disorder, recurrent episode, unspecified
	29634	Major depressive affective disorder, recurrent episode, severe, specified as with psychotic behavior
	29626	Major depressive affective disorder, single episode, in full remission
	29625	Major depressive affective disorder, single episode, in partial or unspecified remission
	29621	Major depressive affective disorder, single episode, mild
	29622	Major depressive affective disorder, single episode, moderate
	29623	Major depressive affective disorder, single episode, severe, without mention of psychotic behavior
	29620	Major depressive affective disorder, single episode, unspecified
	29624	Major depressive affective disorder, single episode, severe, specified as with psychotic behavior
	311	Depressive disorder, not elsewhere classified
	3004	Dysthymic disorder
	6254	Premenstrual tension syndromes

Psychoses	ICD9	ICD9 Description
	29500	Simple type schizophrenia, unspecified
	29501	Simple type schizophrenia, subchronic
	29502	Simple type schizophrenia, chronic
	29503	Simple type schizophrenia, subchronic with acute exacerbation
	29504	Simple type schizophrenia, chronic with acute exacerbation
	29505	Simple type schizophrenia, in remission

© 2020 Kimmel SD et al. *JAMA Network Open.*

	29550	Latent schizophrenia, unspecified
	29551	Latent schizophrenia, subchronic
	29552	Latent schizophrenia, chronic
	29553	Latent schizophrenia, subchronic with acute exacerbation
	29554	Latent schizophrenia, chronic with acute exacerbation
	29555	Latent schizophrenia, in remission
	29580	Other specified types of schizophrenia, unspecified
	29581	Other specified types of schizophrenia, subchronic
	29582	Other specified types of schizophrenia, chronic
	29583	Other specified types of schizophrenia, subchronic with acute exacerbation
	29584	Other specified types of schizophrenia, chronic with acute exacerbation
	29585	Other specified types of schizophrenia, in remission
	29510	Disorganized type schizophrenia, unspecified
	29511	Disorganized type schizophrenia, subchronic
	29512	Disorganized type schizophrenia, chronic
	29513	Disorganized type schizophrenia, subchronic with acute exacerbation
	29514	Disorganized type schizophrenia, chronic with acute exacerbation
	29515	Disorganized type schizophrenia, in remission
	29520	Catatonic type schizophrenia, unspecified
	29521	Catatonic type schizophrenia, subchronic
	29522	Catatonic type schizophrenia, chronic
	29523	Catatonic type schizophrenia, subchronic with acute exacerbation
	29524	Catatonic type schizophrenia, chronic with acute exacerbation
	29525	Catatonic type schizophrenia, in remission
	29530	Paranoid type schizophrenia, unspecified
	29531	Paranoid type schizophrenia, subchronic
	29532	Paranoid type schizophrenia, chronic
	29533	Paranoid type schizophrenia, subchronic with acute exacerbation
	29534	Paranoid type schizophrenia, chronic with acute exacerbation
	29535	Paranoid type schizophrenia, in remission
	29540	Schizophreniform disorder, unspecified
	29541	Schizophreniform disorder, subchronic
	29542	Schizophreniform disorder, chronic
	29543	Schizophreniform disorder, subchronic with acute exacerbation
	29544	Schizophreniform disorder, chronic with acute exacerbation

	29545	Schizophreniform disorder, in remission
	29560	Schizophrenic disorders, residual type, unspecified
	29561	Schizophrenic disorders, residual type, subchronic
	29562	Schizophrenic disorders, residual type, chronic
	29563	Schizophrenic disorders, residual type, subchronic with acute exacerbation
	29564	Schizophrenic disorders, residual type, chronic with acute exacerbation
	29565	Schizophrenic disorders, residual type, in remission
	29670	Schizoaffective disorder, unspecified
	29671	Schizoaffective disorder, subchronic
	29672	Schizoaffective disorder, chronic
	29673	Schizoaffective disorder, subchronic with acute exacerbation
	29674	Schizoaffective disorder, chronic with acute exacerbation
	29675	Schizoaffective disorder, in remission
	29590	Unspecified schizophrenia, unspecified
	29591	Unspecified schizophrenia, subchronic
	29592	Unspecified schizophrenia, chronic
	29593	Unspecified schizophrenia, subchronic with acute exacerbation
	29594	Unspecified schizophrenia, chronic with acute exacerbation
	29595	Unspecified schizophrenia, in remission
	29600	Bipolar I disorder, single manic episode, unspecified
	29610	Manic affective disorder, recurrent episode, unspecified
	29601	Bipolar I disorder, single manic episode, mild
	29611	Manic affective disorder, recurrent episode, mild
	29602	Bipolar I disorder, single manic episode, moderate
	29613	Manic affective disorder, recurrent episode, severe, without mention of psychotic behavior
	29603	Bipolar I disorder, single manic episode, severe, without mention of psychotic behavior
	29612	Manic affective disorder, recurrent episode, moderate
	29604	Bipolar I disorder, single manic episode, severe, specified as with psychotic behavior
	29614	Manic affective disorder, recurrent episode, severe, specified as with psychotic behavior
	29605	Bipolar I disorder, single manic episode, in partial or unspecified remission
	29615	Manic affective disorder, recurrent episode, in partial or unspecified remission

© 2020 Kimmel SD et al. *JAMA Network Open.*

	29606	Bipolar I disorder, single manic episode, in full remission
	29616	Manic affective disorder, recurrent episode, in full remission
	29620	Major depressive affective disorder, single episode, unspecified
	29621	Major depressive affective disorder, single episode, mild
	29622	Major depressive affective disorder, single episode, moderate
	29623	Major depressive affective disorder, single episode, severe, without mention of psychotic behavior
	29624	Major depressive affective disorder, single episode, severe, specified as with psychotic behavior
	29625	Major depressive affective disorder, single episode, in partial or unspecified remission
	29626	Major depressive affective disorder, single episode, in full remission
	29630	Major depressive affective disorder, recurrent episode, unspecified
	29631	Major depressive affective disorder, recurrent episode, mild
	29632	Major depressive affective disorder, recurrent episode, moderate
	29633	Major depressive affective disorder, recurrent episode, severe, without mention of psychotic behavior
	29634	Major depressive affective disorder, recurrent episode, severe, specified as with psychotic behavior
	29635	Major depressive affective disorder, recurrent episode, in partial or unspecified remission
	29636	Major depressive affective disorder, recurrent episode, in full remission
	29640	Bipolar I disorder, most recent episode (or current) manic, unspecified
	29641	Bipolar I disorder, most recent episode (or current) manic, mild
	29642	Bipolar I disorder, most recent episode (or current) manic, moderate
	29643	Bipolar I disorder, most recent episode (or current) manic, severe, without mention of psychotic behavior
	29644	Bipolar I disorder, most recent episode (or current) manic, severe, specified as with psychotic behavior
	29645	Bipolar I disorder, most recent episode (or current) manic, in partial or unspecified remission
	29646	Bipolar I disorder, most recent episode (or current) manic, in full remission

	29650	Bipolar I disorder, most recent episode (or current) depressed, unspecified
	29651	Bipolar I disorder, most recent episode (or current) depressed, mild
	29652	Bipolar I disorder, most recent episode (or current) depressed, moderate
	29653	Bipolar I disorder, most recent episode (or current) depressed, severe, without mention of psychotic behavior
	29654	Bipolar I disorder, most recent episode (or current) depressed, severe, specified as with psychotic behavior
	29655	Bipolar I disorder, most recent episode (or current) depressed, in partial or unspecified remission
	29656	Bipolar I disorder, most recent episode (or current) depressed, in full remission
	29660	Bipolar I disorder, most recent episode (or current) mixed, unspecified
	29660	Bipolar I disorder, most recent episode (or current) mixed, mild
	29660	Bipolar I disorder, most recent episode (or current) mixed, moderate
	29660	Bipolar I disorder, most recent episode (or current) mixed, severe, without mention of psychotic behavior
	29660	Bipolar I disorder, most recent episode (or current) mixed, severe, specified as with psychotic behavior
	29660	Bipolar I disorder, most recent episode (or current) mixed, in partial or unspecified remission
	29660	Bipolar I disorder, most recent episode (or current) mixed, in full remission
	2967	Bipolar I disorder, most recent episode (or current) unspecified
	29680	Bipolar disorder, unspecified
	29681	Atypical manic disorder
	29682	Atypical depressive disorder
	29689	Other bipolar disorders
	29690	Unspecified episodic mood disorder
	29699	Other specified episodic mood disorder
	2970	Paranoid state, simple
	2971	Delusional disorder
	2972	Paraphrenia
	2978	Other specified paranoid states
	2973	Shared psychotic disorder

© 2020 Kimmel SD et al. *JAMA Network Open.*

	2979	Unspecified paranoid state
	2983	Acute paranoid reaction
	2984	Psychogenic paranoid psychosis
	2988	Other and unspecified reactive psychosis
	2980	Depressive type psychosis
	2981	Excitative type psychosis
	2982	Reactive confusion
	2989	Unspecified psychosis
	29910	Childhood disintegrative disorder, current or active state
	29911	Childhood disintegrative disorder, residual state

**Anxiety**	**ICD9**	**ICD9 Description**
	3002	Generalized anxiety disorder
	3009	Other specified anxiety disorder
	3001	Panic disorder
	30000	Unspecified anxiety disorder

e Table 3: ICD-9 Codes used to calculate Elixhauser Scores

HIV	**ICD9**	**ICD9 Description**
	042	Human immunodeficiency virus [HIV] disease
	27910	Immunodeficiency with predominant T-cell defect, unspecified
	V08	Asymptomatic human immunodeficiency virus [HIV] infection status

Liver	**ICD9**	**ICD9 Description**
	07032	Chronic viral hepatitis B without mention of hepatic coma without mention of hepatitis delta
	07054	Chronic hepatitis C without mention of hepatic coma
	4560	Esophageal varices with bleeding
	4561	Esophageal varices without mention of bleeding
	45620	Esophageal varices in diseases classified elsewhere, with bleeding
	45621	Esophageal varices in diseases classified elsewhere, without mention of bleeding
	5710	Alcoholic fatty liver
	5712	Alcoholic cirrhosis of liver

© 2020 Kimmel SD et al. *JAMA Network Open.*

	5713	Alcoholic liver damage, unspecified
	57140	Chronic hepatitis, unspecified
	57141	Chronic persistent hepatitis
	57142	Autoimmune hepatitis
	57149	Other chronic hepatitis
	5715	Cirrhosis of liver without mention of alcohol
	5716	Biliary cirrhosis
	5718	Other chronic nonalcoholic liver disease
	5719	Unspecified chronic liver disease without mention of alcohol
	5723	Portal hypertension
	5728	Other sequelae of chronic liver disease
	V427	Liver replaced by transplant

Congestive Heart Failure	**ICD9**	**ICD9 Description**
	39891	Rheumatic heart failure (congestive)
	40211	Benign hypertensive heart disease with heart failure
	40291	Unspecified hypertensive heart disease with heart failure
	40411	Hyperensive heart and chronic kidney disease with heart failure and with chronic kidney disease stage I
	40413	Hypertensive heart and chronic kidney disease, benign, with heart failure and chronic kidney disease stage V or end
	40493	Hypertensive heart and chronic kidney disease, unspecified, with heart failure and chronic kidney disease stage V or end stage renal disease
	40491	Hypertensive heart and chronic kidney disease, unspecified, with heart failure and with chronic kidney disease stage I through stage IV, or unspecified
	4280	Congestive heart failure, unspecified
	4281	Left heart failure
	42820	Systolic heart failure, unspecified
	42821	Acute systolic heart failure
	42822	Chronic systolic heart failure
	42823	Acute on chronic systolic heart failure
	42830	Diastolic heart failure, unspecified
	42831	Acute diastolic heart failure

© 2020 Kimmel SD et al. *JAMA Network Open.*

	42832	Chronic diastolic heart failure
	42833	Acute on chronic diastolic heart failure
	42840	Combined systolic and diastolic heart failure, unspecified
	42841	Acute combined systolic and diastolic heart failure
	42842	Chronic combined systolic and diastolic heart failure
	42843	Acute on chronic combined systolic and diastolic heart failure
	4289	Heart failure, unspecified

Arrythmia	**ICD9**	**ICD9 Description**
	42610	Atrioventricular block, unspecified
	42611	First degree atrioventricular block
	42612	Mobitz (type) II atrioventricular block
	42613	Other second degree atrioventricular block
	4262	Left bundle branch hemiblock
	4263	Other left bundle branch block
	4264	Right bundle branch block
	42650	Bundle branch block, unspecified
	42651	Right bundle branch block and left posterior fascicular block
	42652	Right bundle branch block and left anterior fascicular block
	42653	Other bilateral bundle branch block
	4266	Other heart block
	4267	Anomalous atrioventricular excitation
	42681	Lown-Ganong-Levine syndrome
	42682	Long QT syndrome
	42689	Other specified conduction disorders
	4270	Paroxysmal supraventricular tachycardia
	4272	Paroxysmal tachycardia, unspecified
	42731	Atrial fibrillation
	42760	Premature beats, unspecified
	4279	Cardiac dysrhythmia, unspecified
	7850	Tachycardia, unspecified
	V4500	Unspecified cardiac device in situ
	V4501	Cardiac pacemaker in situ
	V4502	Automatic implantable cardiac defibrillator in situ

© 2020 Kimmel SD et al. *JAMA Network Open.*

	V4509	Other specified cardiac device in situ
	V5331	Fitting and adjustment of cardiac pacemaker
	V5332	Fitting and adjustment of automatic implantable cardiac defibrillator
	V5339	Fitting and adjustment of other cardiac device

Vascular Disease	**ICD9**	**ICD9 Description**
	09320	Syphilitic endocarditis of valve, unspecified
	3940	Mitral stenosis
	3941	Rheumatic mitral insufficiency
	3942	Mitral stenosis with insufficiency
	3949	Other and unspecified mitral valve diseases
	3950	Rheumatic aortic stenosis
	3951	Rheumatic aortic insufficiency
	3952	Rheumatic aortic stenosis with insufficiency
	3959	Other and unspecified rheumatic aortic diseases
	3960	Mitral valve stenosis and aortic valve stenosis
	3961	Mitral valve stenosis and aortic valve insufficiency
	3962	Mitral valve insufficiency and aortic valve stenosis
	3963	Mitral valve insufficiency and aortic valve insufficiency
	3968	Multiple involvement of mitral and aortic valves
	3969	Mitral and aortic valve diseases, unspecified
	3970	Diseases of tricuspid valve
	3971	Rheumatic diseases of pulmonary valve
	4240	Mitral valve disorders
	4241	Aortic valve disorders
	4242	Diseases of tricuspid valve
	4243	Tricuspid valve disorders, specified as nonrheumatic
	42490	Endocarditis in diseases classified elsewhere
	42491	Acute and subacute infective endocarditis in diseases classified elsewhere
	7463	Congenital stenosis of aortic valve
	7464	Congenital insufficiency of aortic valve
	7465	Congenital mitral stenosis
	7466	Congenital mitral insufficiency
	V422	Heart valve replaced by transplant
	V433	Heart valve replaced by other means

© 2020 Kimmel SD et al. *JAMA Network Open.*

Pulmonary/Circulatory Disease	**ICD9**	**ICD9 Description**
	4160	Primary pulmonary hypertension
	4161	Kyphoscoliotic heart disease
	4162	Chronic pulmonary embolism
	4168	Other chronic pulmonary heart diseases
	4169	Chronic pulmonary heart disease, unspecified
	4179	Other specified diseases of pulmonary circulation

Peripheral Vascular Disease	**ICD9**	**ICD9 Description**
	4400	Atherosclerosis of aorta
	4401	Atherosclerosis of renal artery
	44020	Atherosclerosis of native arteries of the extremities, unspecified
	44021	Atherosclerosis of native arteries of the extremities with intermittent claudication
	44022	Atherosclerosis of native arteries of the extremities with rest pain
	44023	Atherosclerosis of native arteries of the extremities with ulceration
	44024	Atherosclerosis of native arteries of the extremities with gangrene
	44029	Other atherosclerosis of native arteries of the extremities
	44030	Atherosclerosis of unspecified bypass graft of the extremities
	44031	Atherosclerosis of autologous vein bypass graft of the extremities
	44032	Atherosclerosis of nonautologous biological bypass graft of the extremities
	4404	Chronic total occlusion of artery of the extremities
	4408	Atherosclerosis of other specified arteries
	4409	Generalized and unspecified atherosclerosis
	4412	Thoracic aneurysm without mention of rupture
	4414	Abdominal aneurysm without mention of rupture
	4417	Thoracoabdominal aneurysm, without mention of rupture
	4419	Aortic aneurysm of unspecified site without mention of rupture
	4431	Thromboangiitis obliterans [Buerger's disease]
	44321	Dissection of carotid artery
	44322	Dissection of iliac artery

© 2020 Kimmel SD et al. *JAMA Network Open.*

	44323	Dissection of renal artery
	44324	Dissection of vertebral artery
	44329	Dissection of other artery
	44381	Peripheral angiopathy in diseases classified elsewhere
	44382	Erythromelalgia
	44389	Other specified peripheral vascular diseases
	4439	Peripheral vascular disease, unspecified
	4471	Stricture of artery
	5571	Chronic vascular insufficiency of intestine
	5579	Unspecified vascular insufficiency of intestine
	V434	Blood vessel replaced by other means

Hypertension, Controlled	**ICD9**	**ICD9 Description**
	4011	Benign essential hypertension
	4010	Malignant essential hypertension
	4019	Unspecified essential hypertension

Hypertension, Uncontrolled	**ICD9**	**ICD9 Description**
	40200	Malignant hypertensive heart disease without heart failure
	40210	Benign hypertensive heart disease without heart failure
	40211	Benign hypertensive heart disease with heart failure
	40201	Malignant hypertensive heart disease with heart failure
	40291	Unspecified hypertensive heart disease with heart failure
	40290	Unspecified hypertensive heart disease without heart failure
	40401	Hypertensive heart and chronic kidney disease, malignant, with heart failure and with chronic kidney disease stage I through stage IV, or unspecified
	40411	Hypertensive heart and chronic kidney disease, benign, with heart failure and with chronic kidney disease stage I through stage IV, or unspecified
	40491	Hypertensive heart and chronic kidney disease, unspecified, with heart failure and with chronic kidney disease stage I through stage IV, or unspecified
	40501	Malignant renovascular hypertension
	40511	Benign renovascular hypertension
	40591	Unspecified renovascular hypertension
	40509	Other malignant secondary hypertension
	40519	Other benign secondary hypertension

© 2020 Kimmel SD et al. *JAMA Network Open.*

	40599	Other unspecified secondary hypertension

Paralysis	**ICD9**	**ICD9 Description**
	34200	Flaccid hemiplegia and hemiparesis affecting unspecified side
	34210	Spastic hemiplegia and hemiparesis affecting unspecified side
	34290	Hemiplegia, unspecified, affecting unspecified side
	34291	Hemiplegia, unspecified, affecting dominant side
	34292	Hemiplegia, unspecified, affecting nondominant side
	34400	Quadriplegia, unspecified
	34409	Other quadriplegia
	34401	Quadriplegia, C1-C4, complete
	34402	Quadriplegia, C1-C4, incomplete
	34403	Quadriplegia, C5-C7, complete
	34404	Quadriplegia, C5-C7, incomplete
	3441	Paraplegia
	3442	Diplegia of upper limbs
	34430	Monoplegia of lower limb affecting unspecified side
	34431	Monoplegia of lower limb affecting dominant side
	34432	Monoplegia of lower limb affecting nondominant side
	34440	Monoplegia of upper limb affecting unspecified side
	34441	Monoplegia of upper limb affecting dominant side
	34442	Monoplegia of upper limb affecting nondominant sde
	3445	Unspecified monoplegia
	35560	Cauda equina syndrome without mention of neurogenic bladder
	34461	Cauda equina syndrome with neurogenic bladder
	34481	Locked-in state
	34489	Other specified paralytic syndrome
	3449	Paralysis, unspecified

Neurologic Disease	**ICD9**	**ICD9 Description**
	3319	Cerebral degeneration, unspecified
	3320	Paralysis agitans
	3334	Huntington's chorea
	3335	Other choreas
	3340	Friedreich's ataxia
	3343	Other cerebellar ataxia
	3341	Hereditary spastic paraplegia
	3342	Primary cerebellar degeneration

© 2020 Kimmel SD et al. *JAMA Network Open.*

	3344	Cerebellar ataxia in diseases classified elsewhere
	3348	Other spinocerebellar diseases
	3349	Spinocerebellar disease, unspecified
	3350	Werdnig-Hoffmann disease
	33510	Spinal muscular atrophy, unspecified
	3359	Anterior horn cell disease, unspecified
	33511	Kugelberg-Welander disease
	33519	Other spinal muscular atrophy
	33523	Pseudobulbar palsy
	3358	Other anterior horn cell diseases
	33520	Amyotrophic lateral sclerosis
	33521	Progressive muscular atrophy
	33522	Progressive bulbar palsy
	33524	Primary lateral sclerosis
	33529	Other motor neuron disease
	340	Multiple Sclerosis
	3411	Schilder's disease
	34120	Acute (transverse) myelitis NOS
	34121	Acute (transverse) myelitis in conditions classified elsewhere
	34122	Idiopathic transverse myelitis
	3418	Other demyelinating diseases of central nervous system
	3419	Demyelinating disease of central nervous system, unspecified
	34590	Epilepsy Unspecified
	3481	Anoxic brain damage
	34830	Encephalopathy, not elsewhere classified
	78031	Febrile convulsions (simple), unspecified
	78032	Complex febrile convulsions
	78033	Post traumatic seizures
	78039	Other convulsions
	7843	Aphasia

Chronic Pulmonary Disease	**ICD9**	**ICD9 Description**
	490	Bronchitis, not specified as acute or chronic
	4910	Simple chronic bronchiti
	4911	Mucopurulent chronic bronchitis
	49120	Obstructive chronic bronchitis without exacerbation
	49121	Obstructive chronic bronchitis with (acute) exacerbation
	49122	Obstructive chronic bronchitis with acute bronchitis

© 2020 Kimmel SD et al. *JAMA Network Open.*

	4918	Other chronic bronchitis
	4919	Unspecified chronic bronchitis
	4920	Emphysematous bleb
	4928	Other emphysema
	49300	Extrinsic asthma, unspecified
	49310	Intrinsic asthma, unspecified
	49301	Extrinsic asthma with status asthmaticus
	49311	Intrinsic asthma with status asthmaticus
	49302	Extrinsic asthma with (acute) exacerbation
	49312	Intrinsic asthma with (acute) exacerbation
	49320	Chronic obstructive asthma, unspecified
	49321	Chronic obstructive asthma with status asthmaticus
	49322	Chronic obstructive asthma with (acute) exacerbation
	49381	Exercise induced bronchospasm
	49382	Cough variant asthma
	49390	Asthma,unspecified type, unspecified
	49391	Asthma, unspecified type, with status asthmaticus
	4940	Bronchiectasis without acute exacerbation
	4941	Bronchiectasis with acute exacerbation
	4950	Farmers' lung
	4951	Bagassosis
	4952	Bird-fanciers' lung
	4953	Suberosis
	4954	Malt workers' lung
	4955	Mushroom workers' lung
	4956	Maple bark-strippers' lung
	4957	"Ventilation" pneumonitis
	4958	Other specified allergic alveolitis and pneumonitis
	4959	Unspecified allergic alveolitis and pneumonitis
	496	Chronic airway obstruction, not elsewhere classified
	500	Coal workers' pneumoconiosis
	501	Asbestosis
	502	Pneumoconiosis due to other silica or silicates
	503	Pneumoconiosis due to other inorganic dust
	504	Pneumonopathy due to inhalation of other dus
	505	Pneumoconiosis, unspecified

© 2020 Kimmel SD et al. *JAMA Network Open.*

	5064	Chronic respiratory conditions due to fumes and vapors

Diabetes, Controlled	**ICD9**	**ICD9 Description**
	25000	Diabetes mellitus without mention of complication, type II or unspecified type, not stated as uncontrolled
	25001	Diabetes mellitus without mention of complication, type I [juvenile type], not stated as uncontrolled
	25002	Diabetes mellitus without mention of complication, type II or unspecified type, uncontrolled
	25003	Diabetes mellitus without mention of complication, type I [juvenile type], uncontrolled
	25010	Diabetes with ketoacidosis, type II or unspecified type, not stated as uncontrolled
	25011	Diabetes with ketoacidosis, type I [juvenile type], not stated as uncontrolled
	25012	Diabetes with ketoacidosis, type II or unspecified type, uncontrolled
	25013	Diabetes with ketoacidosis, type I [juvenile type], uncontrolled
	25020	Diabetes with hyperosmolarity, type II or unspecified type, not stated as uncontrolled
	25021	Diabetes with hyperosmolarity, type I [juvenile type], not stated as uncontrolled
	25022	Diabetes with hyperosmolarity, type II or unspecified type, uncontrolled
	25023	Diabetes with hyperosmolarity, type I [juvenile type], uncontrolled
	25030	Diabetes with other coma, type II or unspecified type, not stated as uncontrolled
	25031	Diabetes with other coma, type I [juvenile type], not stated as uncontrolled
	25032	Diabetes with other coma, type II or unspecified type, uncontrolled
	25033	Diabetes with other coma, type I [juvenile type], uncontrolled

Diabetes, Uncontrolled	**ICD9**	**ICD9 Description**
	25040	Diabetes with renal manifestations, type II or unspecified type, not stated as uncontrolled
	24041	Diabetes with renal manifestations, type I [juvenile type], not stated as uncontrolled

© 2020 Kimmel SD et al. *JAMA Network Open.*

	25042	Diabetes with renal manifestations, type II or unspecified type, uncontrolled
	25043	Diabetes with renal manifestations, type I [juvenile type], uncontrolled
	25050	Diabetes with ophthalmic manifestations, type II or unspecified type, not stated as uncontrolled
	25051	Diabetes with ophthalmic manifestations, type I [juvenile type], not stated as uncontrolled
	25060	Diabetes with neurological manifestations, type II or unspecified type, not stated as uncontrolled
	25061	Diabetes with neurological manifestations, type I [juvenile type], not stated as uncontrolled
	25063	Diabetes with neurological manifestations, type I [juvenile type], uncontrolled
	25062	Diabetes with neurological manifestations, type II or unspecified type, uncontrolled
	25070	Diabetes with peripheral circulatory disorders, type II or unspecified type, not stated as uncontrolled
	25071	Diabetes with peripheral circulatory disorders, type I [juvenile type], not stated as uncontrolled
	25072	Diabetes with peripheral circulatory disorders, type II or unspecified type, uncontrolled
	25073	Diabetes with peripheral circulatory disorders, type I [juvenile type], uncontrolled
	25090	Diabetes with unspecified complication, type II or unspecified type, not stated as uncontrolled
	25091	Diabetes with unspecified complication, type I [juvenile type], not stated as uncontrolled
	25092	Diabetes with unspecified complication, type II or unspecified type, uncontrolled
	25093	Diabetes with unspecified complication, type I [juvenile type], uncontrolled

Hypothyroidism	**ICD9**	**ICD9 Description**
	243	Congenital hypothyroidism
	2440	Postsurgical hypothyroidism
	2441	Other postablative hypothyroidism
	2442	Iodine hypothyroidism

© 2020 Kimmel SD et al. *JAMA Network Open.*

	2448	Other specified acquired hypothyroidism
	2449	Unspecified acquired hypothyroidism

Renal Failure	**ICD9**	**ICD9 Description**
	40311	Hypertensive chronic kidney disease, benign, with chronic kidney disease stage V or end stage renal disease
	40391	Hypertensive chronic kidney disease, unspecified, with chronic kidney disease stage V or end stage renal disease
	40412	Hypertensive heart and chronic kidney disease, benign, without heart failure and with chronic kidney disease stage V or end stage renal disease
	40492	Hypertensive heart and chronic kidney disease, unspecified, without heart failure and with chronic kidney disease stage V or end stage renal disease
	5851	Chronic kidney disease, Stage I
	5852	Chronic kidney disease, Stage II (mild)
	5853	Chronic kidney disease, Stage III (moderate)
	5854	Chronic kidney disease, Stage IV (severe)
	5855	Chronic kidney disease, Stage V
	5856	End stage renal disease
	5859	Chronic kidney disease, unspecified
	586	Renal Failure, unspecified
	V420	Kidney replaced by transplant
	V4511	Renal dialysis status
	V4512	Noncompliance with renal dialysis
	V560	Encounter for extracorporeal dialysis
	V568	Encounter for other dialysis

Peptic Ulcer	**ICD9**	**ICD9 Description**
	53170	Chronic gastric ulcer without mention of hemorrhage or perforation, without mention of obstruction
	53190	Gastric ulcer, unspecified as acute or chronic, without mention of hemorrhage or perforation, without mention of obstruction
	53270	Chronic duodenal ulcer without mention of hemorrhage or perforation, without mention of obstruction
	53290	Duodenal ulcer, unspecified as acute or chronic, without hemorrhage or perforation, without mention of obstruction

© 2020 Kimmel SD et al. *JAMA Network Open.*

	53370	Chronic peptic ulcer of unspecified site without mention of hemorrhage or perforation, without mention of obstruction
	53390	Peptic ulcer of unspecified site, unspecified as acute or chronic, without mention of hemorrhage or perforation, without mention of obstruction
	53470	Chronic gastrojejunal ulcer without mention of hemorrhage or perforation, without mention of obstruction
	53490	Gastrojejunal ulcer, unspecified as acute or chronic, without mention of hemorrhage or perforation, without mention of obstruction
	V1271	Personal history of peptic ulcer disease

Lymphoma	**ICD9**	**ICD9 Description**
	20000	Reticulosarcoma, unspecified site, extranodal and solid organ sites
	20001	Reticulosarcoma, lymph nodes of head, face, and neck
	20002	Reticulosarcoma, intrathoracic lymph nodes
	20003	Reticulosarcoma, intra-abdominal lymph nodes
	20004	Reticulosarcoma, lymph nodes of axilla and upper limb
	20005	Reticulosarcoma, lymph nodes of inguinal region and lower limb
	20006	Reticulosarcoma, intrapelvic lymph nodes
	20007	Reticulosarcoma, spleen
	20008	Reticulosarcoma, lymph nodes of multiple sites
	20010	Lymphosarcoma, unspecified site, extranodal and solid organ sites
	20011	Lymphosarcoma, lymph nodes of head, face, and neck
	20012	Lymphosarcoma, intrathoracic lymph nodes
	20013	Lymphosarcoma, intra-abdominal lymph nodes
	20014	Lymphosarcoma, lymph nodes of axilla and upper limb
	20015	Lymphosarcoma, lymph nodes of inguinal region and lower limb
	20016	Lymphosarcoma, intrapelvic lymph nodes
	20017	Lymphosarcoma, spleen

© 2020 Kimmel SD et al. *JAMA Network Open.*

	20018	Lymphosarcoma, lymph nodes of multiple sites
	20020	Burkitt's tumor or lymphoma, unspecified site, extranodal and solid organ sites
	20021	Burkitt's tumor or lymphoma, lymph nodes of head, face, and neck
	20022	Burkitt's tumor or lymphoma, intrathoracic lymph nodes
	20023	Burkitt's tumor or lymphoma, intra-abdominal lymph nodes
	20024	Burkitt's tumor or lymphoma, lymph nodes of axilla and upper limb
	20025	Burkitt's tumor or lymphoma, lymph nodes of inguinal region and lower limb
	20026	Burkitt's tumor or lymphoma, intrapelvic lymph nodes
	20027	Burkitt's tumor or lymphoma, spleen
	20028	Burkitt's tumor or lymphoma, lymph nodes of multiple sites
	20030	Marginal zone lymphoma, unspecified site, extranodal and solid organ sites
	20031	Marginal zone lymphoma, lymph nodes of head, face, and neck
	20032	Marginal zone lymphoma, intrathoracic lymph nodes
	20033	Marginal zone lymphoma, intraabdominal lymph nodes
	20034	Marginal zone lymphoma, lymph nodes of axilla and upper limb
	20035	Marginal zone lymphoma, lymph nodes of inguinal region and lower limb
	20036	Marginal zone lymphoma, intrapelvic lymph nodes
	20037	Marginal zone lymphoma, spleen
	20038	Marginal zone lymphoma, lymph nodes of multiple sites
	20040	Mantle cell lymphoma, unspecified site, extranodal and solid organ sites
	20041	Mantle cell lymphoma, lymph nodes of head, face, and neck
		Mantle cell lymphoma, intrathoracic lymph nodes
	20042	Mantle cell lymphoma, intra-abdominal lymph nodes
		Mantle cell lymphoma, lymph nodes of axilla and upper limb
	20043	Mantle cell lymphoma, lymph nodes of inguinal region and lower limb
		Mantle cell lymphoma, intrapelvic lymph nodes
	20044	Mantle cell lymphoma, spleen
		Mantle cell lymphoma, lymph nodes of multiple sites

	20050	Primary central nervous system lymphoma, unspecified site, extranodal and solid organ sites
	20052	Primary central nervous system lymphoma, intrathoracic lymph nodes
	20053	Primary central nervous system lymphoma, intra-abdominal lymph nodes
	20055	Primary central nervous system lymphoma, lymph nodes of inguinal region and lower limb
	20056	Primary central nervous system lymphoma, intrapelvic lymph nodes
	20057	Primary central nervous system lymphoma, spleen
	20058	Primary central nervous system lymphoma, lymph nodes of multiple sites
	20060	Anaplastic large cell lymphoma, unspecified site, extranodal and solid organ sites
	20061	Anaplastic large cell lymphoma, lymph nodes of head, face, and neck
	20062	Anaplastic large cell lymphoma, intrathoracic lymph nodes
	20063	Anaplastic large cell lymphoma, intra-abdominal lymph nodes
	20064	Anaplastic large cell lymphoma, lymph nodes of axilla and upper limb
	20065	Anaplastic large cell lymphoma, lymph nodes of inguinal region and lower limb
	20066	Anaplastic large cell lymphoma, intrapelvic lymph nodes
	20067	Anaplastic large cell lymphoma, spleen
	20068	Anaplastic large cell lymphoma, lymph nodes of multiple sites
	20070	Large cell lymphoma, unspecified site, extranodal and solid organ sites
	20071	Large cell lymphoma, lymph nodes of head, face, and neck
	20072	Large cell lymphoma, intrathoracic lymph nodes
	20073	Large cell lymphoma, intra-abdominal lymph nodes
	20074	Large cell lymphoma, lymph nodes of axilla and upper limb
	20075	Large cell lymphoma, lymph nodes of inguinal region and lower limb
	20076	Large cell lymphoma, intrapelvic lymph nodes
	20077	Large cell lymphoma, spleen

© 2020 Kimmel SD et al. *JAMA Network Open.*

	20078	Large cell lymphoma, lymph nodes of multiple sites
	20080	Other named variants of lymphosarcoma and reticulosarcoma, unspecified site, extranodal and solid organ sites
	20081	Other named variants of lymphosarcoma and reticulosarcoma, lymph nodes of head, face, and neck
	20082	Other named variants of lymphosarcoma and reticulosarcoma,intrathoracic lymph nodes
	20083	Other named variants of lymphosarcoma and reticulosarcoma, intra-abdominal lymph nodes
	20084	Other named variants of lymphosarcoma and reticulosarcoma, lymph nodes of axilla and upper limb
	20085	Other named variants of lymphosarcoma and reticulosarcoma, lymph nodes of inguinal region and lower limb
	20086	Other named variants of lymphosarcoma and reticulosarcoma, intrapelvic lymph nodes
	20087	Other named variants of lymphosarcoma and reticulosarcoma, spleen
	20088	Other named variants of lymphosarcoma and reticulosarcoma, lymph nodes of multiple sites
	20100	Hodgkin's paragranuloma, unspecified site, extranodal and solid organ sites
	20101	Hodgkin's paragranuloma, lymph nodes of head, face, and neck
	20102	Hodgkin's paragranuloma, intrathoracic lymph nodes
	20103	Hodgkin's paragranuloma, intra-abdominal lymph nodes
	20104	Hodgkin's paragranuloma, lymph nodes of axilla and upper limb
	20105	Hodgkin's paragranuloma, lymph nodes of inguinal region and lower limb
	20106	Hodgkin's paragranuloma, intrapelvic lymph nodes
	20107	Hodgkin's paragranuloma, spleen
	20108	Hodgkin's paragranuloma, lymph nodes of multiple sites
	20110	Hodgkin's granuloma, unspecified site, extranodal and solid organ sites
	20120	Hodgkin's sarcoma, unspecified site, extranodal and solid organ sites
	20111	Hodgkin's granuloma, lymph nodes of head, face, and neck
	20121	Hodgkin's sarcoma, lymph nodes of head, face, and neck
	20112	Hodgkin's granuloma, intrathoracic lymph nodes
	20122	Hodgkin's sarcoma, intrathoracic lymph nodes

© 2020 Kimmel SD et al. *JAMA Network Open.*

	20113	Hodgkin's granuloma, intra-abdominal lymph nodes
	20123	Hodgkin's sarcoma, intra-abdominal lymph nodes
	20114	Hodgkin's granuloma, lymph nodes of axilla and upper limb
	20124	Hodgkin's sarcoma, lymph nodes of axilla and upper limb
	20115	Hodgkin's granuloma, lymph nodes of inguinal region and lower limb
	20125	Hodgkin's sarcoma, lymph nodes of inguinal region and lower limb
	20116	Hodgkin's granuloma, intrapelvic lymph nodes
	20126	Hodgkin's sarcoma, intrapelvic lymph nodes
	20117	Hodgkin's granuloma, spleen
	20127	Hodgkin's sarcoma, spleen
	20118	Hodgkin's granuloma, lymph nodes of multiple sites
	20128	Hodgkin's sarcoma, lymph nodes of multiple sites
	20140	Hodgkin's disease, lymphocytic-histiocytic predominance, unspecified site, extranodal and solid organ sites
	20141	Hodgkin's disease, lymphocytic-histiocytic predominance, lymph nodes of head, face, and neck
	20142	Hodgkin's disease, lymphocytic-histiocytic predominance, intrathoracic lymph nodes
	20143	Hodgkin's disease, lymphocytic-histiocytic predominance, intra-abdominal lymph nodes
	20144	Hodgkin's disease, lymphocytic-histiocytic predominance, lymph nodes of axilla and upper limb
	20145	Hodgkin's disease, lymphocytic-histiocytic predominance, lymph nodes of inguinal region and lower limb
	20146	Hodgkin's disease, lymphocytic-histiocytic predominance, intrapelvic lymph nodes
	20147	Hodgkin's disease, lymphocytic-histiocytic predominance, spleen
	20148	Hodgkin's disease, lymphocytic-histiocytic predominance, lymph nodes of multiple sites
	20150	Hodgkin's disease, nodular sclerosis, unspecified site, extranodal and solid organ sites
	20151	Hodgkin's disease, nodular sclerosis, lymph nodes of head, face, and neck
	20152	Hodgkin's disease, nodular sclerosis, intrathoracic lymph nodes
	20153	Hodgkin's disease, nodular sclerosis, intra-abdominal lymph nodes

© 2020 Kimmel SD et al. *JAMA Network Open.*

	20154	Hodgkin's disease, nodular sclerosis, lymph nodes of axilla and upper limb
	20155	Hodgkin's disease, nodular sclerosis, lymph nodes of inguinal region and lower limb
	20156	Hodgkin's disease, nodular sclerosis, intrapelvic lymph nodes
	20157	Hodgkin's disease, nodular sclerosis, spleen
	20158	Hodgkin's disease, nodular sclerosis, lymph nodes of multiple sites
	20160	Hodgkin's disease, mixed cellularity, unspecified site, extranodal and solid organ sites
	20161	Hodgkin's disease, mixed cellularity, lymph nodes of head, face, and neck
	20162	Hodgkin's disease, mixed cellularity, intrathoracic lymph nodes
	20163	Hodgkin's disease, mixed cellularity, intra-abdominal lymph nodes
	20164	Hodgkin's disease, mixed cellularity, lymph nodes of axilla and upper limb
	20165	Hodgkin's disease, mixed cellularity, lymph nodes of inguinal region and lower limb
	20166	Hodgkin's disease, mixed cellularity, intrapelvic lymph nodes
	20167	Hodgkin's disease, mixed cellularity, spleen
	20168	Hodgkin's disease, mixed cellularity, lymph nodes of multiple sites
	20170	Hodgkin's disease, lymphocytic depletion, unspecified site, extranodal and solid organ sites
	20171	Hodgkin's disease, lymphocytic depletion, lymph nodes of head, face, and neck
	20172	Hodgkin's disease, lymphocytic depletion, intrathoracic lymph nodes
	20173	Hodgkin's disease, lymphocytic depletion, intra-abdominal lymph nodes
	20174	Hodgkin's disease, lymphocytic depletion, lymph nodes of axilla and upper limb
	20175	Hodgkin's disease, lymphocytic depletion, lymph nodes of inguinal region and lower limb
	20176	Hodgkin's disease, lymphocytic depletion, intrapelvic lymph nodes
	20177	Hodgkin's disease, lymphocytic depletion, spleen

	20178	Hodgkin's disease, lymphocytic depletion, lymph nodes of multiple sites
	20190	Hodgkin's disease, unspecified type, unspecified site, extranodal and solid organ sites
	20191	Hodgkin's disease, unspecified type, lymph nodes of head, face, and neck
	20192	Hodgkin's disease, unspecified type, intrathoracic lymph nodes
	20193	Hodgkin's disease, unspecified type, intra-abdominal lymph nodes
	20194	Hodgkin's disease, unspecified type, lymph nodes of axilla and upper limb
	20195	Hodgkin's disease, unspecified type, lymph nodes of inguinal region and lower limb
	20196	Hodgkin's disease, unspecified type, intrapelvic lymph nodes
	20197	Hodgkin's disease, unspecified type, spleen
	20198	Hodgkin's disease, unspecified type, lymph nodes of multiple sites
	20200	Nodular lymphoma, unspecified site, extranodal and solid organ sites
	20201	Nodular lymphoma, lymph nodes of head, face, and nec
	20202	Nodular lymphoma, intrathoracic lymph nodes
	20203	Nodular lymphoma, intra-abdominal lymph nodes
	20204	Nodular lymphoma, lymph nodes of axilla and upper limb
	20205	Nodular lymphoma, lymph nodes of inguinal region and lower limb
	20206	Nodular lymphoma, intrapelvic lymph nodes
	20207	Nodular lymphoma, spleen
	20208	Nodular lymphoma, lymph nodes of multiple sites
	20210	Mycosis fungoides, unspecified site, extranodal and solid organ sites
	20211	Mycosis fungoides, lymph nodes of head, face, and neck
	20212	Mycosis fungoides, intrathoracic lymph nodes
	20213	Mycosis fungoides, intra-abdominal lymph nodes
	20214	Mycosis fungoides, lymph nodes of axilla and upper limb
	20215	Mycosis fungoides, lymph nodes of inguinal region and lower limb
	20216	Mycosis fungoides, intrapelvic lymph nodes
	20217	Mycosis fungoides, spleen

© 2020 Kimmel SD et al. *JAMA Network Open.*

	20218	Mycosis fungoides, lymph nodes of multiple sites
	20220	Sezary's disease, unspecified site, extranodal and solid organ sites
	20221	Sezary's disease, lymph nodes of head, face, and neck
	20222	Sezary's disease, intrathoracic lymph nodes
	20223	Sezary's disease, intra-abdominal lymph nodes
	20224	Sezary's disease, lymph nodes of axilla and upper limb
	20225	Sezary's disease, lymph nodes of inguinal region and lower limb
	20226	Sezary's disease, intrapelvic lymph nodes
	20227	Sezary's disease, spleen
	20228	Sezary's disease, lymph nodes of multiple sites
	20230	Malignant histiocytosis, unspecified site, extranodal and solid organ sites
	20231	Malignant histiocytosis, lymph nodes of head, face, and neck
	20232	Malignant histiocytosis, intrathoracic lymph nodes
	20233	Malignant histiocytosis, intra-abdominal lymph nodes
	20234	Malignant histiocytosis, lymph nodes of axilla and upper limb
	20235	Malignant histiocytosis, lymph nodes of inguinal region and lower limb
	20236	Malignant histiocytosis, intrapelvic lymph nodes
	20237	Malignant histiocytosis, spleen
	20238	Malignant histiocytosis, lymph nodes of multiple sites
	20250	Letterer-siwe disease, unspecified site, extranodal and solid organ sites
	20251	Letterer-siwe disease, lymph nodes of head, face, and neck
	20252	Letterer-siwe disease, intrathoracic lymph nodes
	20253	Letterer-siwe disease, intra-abdominal lymph nodes
	20254	Letterer-siwe disease, lymph nodes of axilla and upper limb
	20255	Letterer-siwe disease, lymph nodes of inguinal region and lower limb
	20256	Letterer-siwe disease, intrapelvic lymph nodes
	20257	Letterer-siwe disease, spleen

© 2020 Kimmel SD et al. *JAMA Network Open.*

	20258	Letterer-siwe disease, lymph nodes of multiple sites
	20260	Malignant mast cell tumors, unspecified site, extranodal and solid organ sites
	20261	Malignant mast cell tumors, lymph nodes of head, face, and neck
	20262	Malignant mast cell tumors, intrathoracic lymph nodes
	20263	Malignant mast cell tumors, intra-abdominal lymph nodes
	20264	Malignant mast cell tumors, lymph nodes of axilla and upper limb
	20265	Malignant mast cell tumors, lymph nodes of inguinal region and lower limb
	20266	Malignant mast cell tumors, intrapelvic lymph nodes
	20267	Malignant mast cell tumors, spleen
	20268	Malignant mast cell tumors, lymph nodes of multiple sites
	20270	Peripheral T cell lymphoma, unspecified site, extranodal and solid organ sites
	20271	Peripheral T cell lymphoma, lymph nodes of head, face, and neck
	20272	Peripheral T cell lymphoma, intrathoracic lymph nodes
	20273	Peripheral T cell lymphoma, intra-abdominal lymph nodes
	20274	Peripheral T cell lymphoma, lymph nodes of axilla and upper limb
	20275	Peripheral T cell lymphoma, lymph nodes of inguinal region and lower limb
	20276	Peripheral T cell lymphoma, intrapelvic lymph nodes
	20277	Peripheral T cell lymphoma, spleen
	20278	Peripheral T cell lymphoma, lymph nodes of multiple sites
	20280	Other malignant lymphomas, unspecified site, extranodal and solid organ sites
	20281	Other malignant lymphomas, lymph nodes of head, face, and neck
	20282	Other malignant lymphomas, intrathoracic lymph nodes
	20283	Other malignant lymphomas, intra-abdominal lymph nodes

© 2020 Kimmel SD et al. *JAMA Network Open.*

	20284	Other malignant lymphomas, lymph nodes of axilla and upper limb
	20285	Other malignant lymphomas, lymph nodes of inguinal region and lower limb
	20286	Other malignant lymphomas, intrapelvic lymph nodes
	20287	Other malignant lymphomas, spleen
	20288	Other malignant lymphomas, lymph nodes of multiple sites
	20290	Other and unspecified malignant neoplasms of lymphoid and histiocytic tissue, unspecified site, extranodal and solid organ sites
	20292	Other and unspecified malignant neoplasms of lymphoid and histiocytic tissue, intrathoracic lymph nodes
	20293	Other and unspecified malignant neoplasms of lymphoid and histiocytic tissue, intra-abdominal lymph nodes
	20294	Other and unspecified malignant neoplasms of lymphoid and histiocytic tissue, lymph nodes of axilla and upper limb
	20295	Other and unspecified malignant neoplasms of lymphoid and histiocytic tissue, lymph nodes of inguinal region and lower lim
	20296	Other and unspecified malignant neoplasms of lymphoid and histiocytic tissue, intrapelvic lymph nodes
	20297	Other and unspecified malignant neoplasms of lymphoid and histiocytic tissue, spleen
	20298	Other and unspecified malignant neoplasms of lymphoid and histiocytic tissue, lymph nodes of multiple sites
	20300	Multiple myeloma, without mention of having achieved remission
	20301	Multiple myeloma, in remission
	20302	Multiple myeloma, in relapse
	20380	Other immunoproliferative neoplasms, without mention of having achieved remission
	20381	Other immunoproliferative neoplasms, in remission
	20382	Other immunoproliferative neoplasms, in relapse
	2386	Neoplasm of uncertain behavior of plasma cells
	2733	Macroglobulinemia
	V1071	Personal history of lymphosarcoma and reticulosarcoma
	V1072	Personal history of hodgkin's disease

© 2020 Kimmel SD et al. *JAMA Network Open.*

	V1079	Personal history of other lymphatic and hematopoietic neoplasms

Metastatic Disease	**ICD9**	**ICD9 Description**
	1960	Secondary and unspecified malignant neoplasm of lymph nodes of head, face, and neck
	1961	Secondary and unspecified malignant neoplasm of intrathoracic lymph nodes
	1962	Secondary and unspecified malignant neoplasm of intra-abdominal lymph nodes
	1963	Secondary and unspecified malignant neoplasm of lymph nodes of axilla and upper limb
	1965	Secondary and unspecified malignant neoplasm of lymph nodes of inguinal region and lower limb
	1966	Secondary and unspecified malignant neoplasm of intrapelvic lymph nodes
	1968	Secondary and unspecified malignant neoplasm of lymph nodes of multiple sites
	1969	Secondary and unspecified malignant neoplasm of lymph nodes, site unspecified
	1970	Secondary malignant neoplasm of lung
	1971	Secondary malignant neoplasm of mediastinum
	1972	Secondary malignant neoplasm of pleura
	1973	Secondary malignant neoplasm of other respiratory organs
	1974	Secondary malignant neoplasm of small intestine including duodenum
	1975	Secondary malignant neoplasm of large intestine and rectum
	1976	Secondary malignant neoplasm of retroperitoneum and peritoneum
	1977	Malignant neoplasm of liver, secondary
	1978	Secondary malignant neoplasm of other digestive organs and spleen
	1980	Secondary malignant neoplasm of kidney
	1981	Secondary malignant neoplasm of other urinary organs
	1982	Secondary malignant neoplasm of skin
	1983	Secondary malignant neoplasm of brain and spinal cord
	1984	Secondary malignant neoplasm of other parts of nervous system

© 2020 Kimmel SD et al. *JAMA Network Open.*

	1985	Secondary malignant neoplasm of bone and bone marrow
	1986	Secondary malignant neoplasm of ovary
	1987	Secondary malignant neoplasm of adrenal gland
	19881	Secondary malignant neoplasm of breast
	19882	Secondary malignant neoplasm of genital organs
	19889	Secondary malignant neoplasm of other specified sites
	1990	Disseminated malignant neoplasm without specification of site
	1991	Other malignant neoplasm without specification of site
	1992	Malignant neoplasm associated with transplant organ

Solid Tumor	**ICD9**	**ICD9 Description**
	1400	Malignant neoplasm of upper lip, vermilion border
	1401	Malignant neoplasm of lower lip, vermilion border
	1402	Malignant neoplasm of upper lip, inner aspect
	1404	Malignant neoplasm of lower lip, inner aspect
	1405	Malignant neoplasm of lip, unspecified, inner aspect
	1406	Malignant neoplasm of commissure of lip
	1408	Malignant neoplasm of other sites of lip
	1409	Malignant neoplasm of lip, unspecified, vermilion border
	1410	Malignant neoplasm of base of tongue
	1411	Malignant neoplasm of dorsal surface of tongue
	1412	Malignant neoplasm of tip and lateral border of tongue
	1413	Malignant neoplasm of ventral surface of tongue
	1414	Malignant neoplasm of anterior two-thirds of tongue, part unspecified
	1415	Malignant neoplasm of junctional zone of tongue
	1416	Malignant neoplasm of lingual tonsil
	1418	Malignant neoplasm of other sites of tongue
	1419	Malignant neoplasm of tongue, unspecified
	1420	Malignant neoplasm of parotid gland
	1421	Malignant neoplasm of submandibular gland
	1422	Malignant neoplasm of sublingual gland
	1428	Malignant neoplasm of other major salivary glands
	1429	Malignant neoplasm of salivary gland, unspecified
	1430	Malignant neoplasm of upper gum
	1431	Malignant neoplasm of lower gum
	1438	Malignant neoplasm of other sites of gum
	1439	Malignant neoplasm of gum, unspecified

© 2020 Kimmel SD et al. *JAMA Network Open.*

	1440	Malignant neoplasm of anterior portion of floor of mouth
	1441	Malignant neoplasm of lateral portion of floor of mouth
	1448	Malignant neoplasm of other sites of floor of mouth
	1449	Malignant neoplasm of floor of mouth, part unspecified
	1450	Malignant neoplasm of cheek mucosa
	1451	Malignant neoplasm of vestibule of mouth
	1451	Malignant neoplasm of hard palate
	1451	Malignant neoplasm of soft palat
	1451	Malignant neoplasm of uvula
	1451	Malignant neoplasm of palate, unspecified
	1451	Malignant neoplasm of retromolar area
	1458	Malignant neoplasm of other specified parts of mouth
	1459	Malignant neoplasm of mouth, unspecified
	1460	Malignant neoplasm of tonsil
	1461	Malignant neoplasm of tonsillar fossa
	1462	Malignant neoplasm of tonsillar pillars (anterior) (posterior)
	1463	Malignant neoplasm of vallecula epiglottica
	1464	Malignant neoplasm of anterior aspect of epiglottis
	1465	Malignant neoplasm of junctional region of oropharynx
	1466	Malignant neoplasm of lateral wall of oropharynx
	1467	Malignant neoplasm of posterior wall of oropharynx
	1468	Malignant neoplasm of other specified sites of oropharynx
	1469	Malignant neoplasm of oropharynx, unspecified site
	1470	Malignant neoplasm of superior wall of nasopharynx
	1470	Malignant neoplasm of posterior wall of nasopharynx
	1470	Malignant neoplasm of lateral wall of nasopharynx
	1470	Malignant neoplasm of anterior wall of nasopharynx
	1478	Malignant neoplasm of other specified sites of nasopharynx
	1479	Malignant neoplasm of nasopharynx, unspecified site
	1480	Malignant neoplasm of postcricoid region of hypopharynx
	1481	Malignant neoplasm of pyriform sinus
	1482	Malignant neoplasm of aryepiglottic fold, hypopharyngeal aspect
	1483	Malignant neoplasm of posterior hypopharyngeal wall
	1488	Malignant neoplasm of other specified sites of hypopharynx
	1489	Malignant neoplasm of hypopharynx, unspecified site
	1490	Malignant neoplasm of pharynx, unspecified
	1491	Malignant neoplasm of waldeyer's ring

© 2020 Kimmel SD et al. *JAMA Network Open.*

	1498	Malignant neoplasm of other sites within the lip and oral cavity
	1499	Malignant neoplasm of ill-defined sites within the lip and oral cavity
	1500	Malignant neoplasm of cervical esophagus
	1503	Malignant neoplasm of upper third of esophagus
	1501	Malignant neoplasm of thoracic esophagus
	1504	Malignant neoplasm of middle third of esophagus
	1502	Malignant neoplasm of abdominal esophagus
	1505	Malignant neoplasm of lower third of esophagus
	1508	Malignant neoplasm of other specified part of esophagus
	1509	Malignant neoplasm of esophagus, unspecified site
	1510	Malignant neoplasm of cardia
	1511	Malignant neoplasm of pylorus
	1512	Malignant neoplasm of pyloric antrum
	1513	Malignant neoplasm of fundus of stomach
	1514	Malignant neoplasm of body of stomach
	1515	Malignant neoplasm of lesser curvature of stomach, unspecified
	1516	Malignant neoplasm of greater curvature of stomach, unspecified
	1518	Malignant neoplasm of other specified sites of stomach
	1519	Malignant neoplasm of stomach, unspecified site
	1520	Malignant neoplasm of duodenum
	1521	Malignant neoplasm of jejunum
	1522	Malignant neoplasm of ileum
	1523	Malignant neoplasm of Meckel's diverticulum
	1528	Malignant neoplasm of other specified sites of small intestine
	1529	Malignant neoplasm of small intestine, unspecified site
	1530	Malignant neoplasm of hepatic flexure
	1531	Malignant neoplasm of transverse colon
	1532	Malignant neoplasm of descending colon
	1533	Malignant neoplasm of sigmoid colon
	1534	Malignant neoplasm of cecum
	1535	Malignant neoplasm of appendix vermiformis
	1536	Malignant neoplasm of ascending colon
	1537	Malignant neoplasm of splenic flexure
	1538	Malignant neoplasm of other specified sites of large intestine
	1539	Malignant neoplasm of colon, unspecified site

© 2020 Kimmel SD et al. *JAMA Network Open.*

	1540	Malignant neoplasm of rectosigmoid junction
	1541	Malignant neoplasm of rectum
	1542	Malignant neoplasm of anal canal
	1543	Malignant neoplasm of anus, unspecified site
	1548	Malignant neoplasm of other sites of rectum, rectosigmoid junction, and anus
	1550	Malignant neoplasm of liver, primary
	1551	Malignant neoplasm of intrahepatic bile ducts
	1552	Malignant neoplasm of liver, not specified as primary or secondary
	1560	Malignant neoplasm of gallbladder
	1561	Malignant neoplasm of extrahepatic bile ducts
	1562	Malignant neoplasm of ampulla of vater
	1568	Malignant neoplasm of other specified sites of gallbladder and extrahepatic bile ducts
	1569	Malignant neoplasm of biliary tract, part unspecified site
	1570	Malignant neoplasm of head of pancreas
	1571	Malignant neoplasm of body of pancreas
	1572	Malignant neoplasm of tail of pancreas
	1573	Malignant neoplasm of pancreatic duct
	1574	Malignant neoplasm of islets of langerhans
	1578	Malignant neoplasm of other specified sites of pancreas
	1579	Malignant neoplasm of pancreas, part unspecified
	1580	Malignant neoplasm of retroperitoneum
	1588	Malignant neoplasm of specified parts of peritoneum
	1589	Malignant neoplasm of peritoneum, unspecified
	1590	Malignant neoplasm of intestinal tract, part unspecified
	1591	Malignant neoplasm of spleen, not elsewhere classified
	1598	Malignant neoplasm of other sites of digestive system and intra-abdominal organs
	1599	Malignant neoplasm of ill-defined sites within the digestive organs and peritoneum
	1600	Malignant neoplasm of nasal cavities
	1601	Malignant neoplasm of auditory tube, middle ear, and mastoid air cells
	1602	Malignant neoplasm of maxillary sinus
	1603	Malignant neoplasm of ethmoidal sinus

© 2020 Kimmel SD et al. *JAMA Network Open.*

	1604	Malignant neoplasm of frontal sinus
	1605	Malignant neoplasm of sphenoidal sinus
	1608	Malignant neoplasm of other accessory sinuses
	1609	Malignant neoplasm of accessory sinus, unspecified
	1610	Malignant neoplasm of glottis
	1611	Malignant neoplasm of supraglottis
	1612	Malignant neoplasm of subglottis
	1613	Malignant neoplasm of laryngeal cartilages
	1618	Malignant neoplasm of other specified sites of larynx
	1619	Malignant neoplasm of larynx, unspecified
	1620	Malignant neoplasm of trachea
	1622	Malignant neoplasm of main bronchus
	1623	Malignant neoplasm of upper lobe, bronchus or lung
	1624	Malignant neoplasm of middle lobe, bronchus or lung
	1625	Malignant neoplasm of lower lobe, bronchus or lung
	1628	Malignant neoplasm of other parts of bronchus or lung
	1629	Malignant neoplasm of bronchus and lung, unspecified
	1630	Malignant neoplasm of parietal pleura
	1631	Malignant neoplasm of visceral pleura
	1638	Malignant neoplasm of other specified sites of pleura
	1639	Malignant neoplasm of pleura, unspecified
	1640	Malignant neoplasm of thymus
	1641	Malignant neoplasm of heart
	1642	Malignant neoplasm of anterior mediastinum
	1642	Malignant neoplasm of posterior mediastinum
	1648	Malignant neoplasm of other parts of mediastinum
	1649	Malignant neoplasm of mediastinum, part unspecified
	1650	Malignant neoplasm of upper respiratory tract, part unspecified
	1658	Malignant neoplasm of other sites within the respiratory system and intrathoracic organs
	1659	Malignant neoplasm of ill-defined sites within the respiratory system

© 2020 Kimmel SD et al. *JAMA Network Open.*

	1700	Malignant neoplasm of bones of skull and face, except mandible
	1701	Malignant neoplasm of mandible
	1702	Malignant neoplasm of vertebral column, excluding sacrum and coccyx
	1703	Malignant neoplasm of ribs, sternum, and clavicle
	1704	Malignant neoplasm of scapula and long bones of upper limb
	1705	Malignant neoplasm of short bones of upper limb
	1706	Malignant neoplasm of pelvic bones, sacrum, and coccyx
	1707	Malignant neoplasm of long bones of lower limb
	1708	Malignant neoplasm of short bones of lower limb
	1709	Malignant neoplasm of bone and articular cartilage, site unspecified
	1710	Malignant neoplasm of connective and other soft tissue of head, face, and neck
	1712	Malignant neoplasm of connective and other soft tissue of upper limb, including shoulder
	1713	Malignant neoplasm of connective and other soft tissue of lower limb, including hip
	1714	Malignant neoplasm of connective and other soft tissue of thorax
	1715	Malignant neoplasm of connective and other soft tissue of abdomen
	1716	Malignant neoplasm of connective and other soft tissue of pelvis
	1717	Malignant neoplasm of connective and other soft tissue of trunk, unspecified
	1718	Malignant neoplasm of other specified sites of connective and other soft tissue
	1719	Malignant neoplasm of connective and other soft tissue, site unspecified
	1720	Malignant melanoma of skin of lip
	1721	Malignant melanoma of skin of eyelid, including canthus
	1723	Malignant melanoma of skin of other and unspecified parts of face
	1724	Malignant melanoma of skin of scalp and neck
	1725	Malignant melanoma of skin of trunk, except scrotum
	1727	Malignant melanoma of skin of lower limb, including hip
	1728	Malignant melanoma of other specified sites of skin
	1729	Melanoma of skin, site unspecified
	1740	Malignant neoplasm of nipple and areola of female breast

© 2020 Kimmel SD et al. *JAMA Network Open.*

	1741	Malignant neoplasm of central portion of female breast
	1742	Malignant neoplasm of upper-inner quadrant of female breast
	1743	Malignant neoplasm of lower-inner quadrant of female breast
	1744	Malignant neoplasm of upper-outer quadrant of female breast
	1745	Malignant neoplasm of lower-outer quadrant of female breast
	1746	Malignant neoplasm of axillary tail of female breast
	1748	Malignant neoplasm of other specified sites of female breast
	1749	Malignant neoplasm of breast (female), unspecified
	1750	Malignant neoplasm of nipple and areola of male breast
	1759	Malignant neoplasm of other and unspecified sites of male breast
	179	Malignant neoplasm of uterus, part unspecified
	1800	Malignant neoplasm of endocervix
	1801	Malignant neoplasm of exocervix
	1808	Malignant neoplasm of other specified sites of cervix
	1809	Malignant neoplasm of cervix uteri, unspecified site
	181	Malignant neoplasm of placenta
	1820	Malignant neoplasm of corpus uteri, except isthmus
	1821	Malignant neoplasm of isthmus
	1828	Malignant neoplasm of other specified sites of body of uterus
	1830	Malignant neoplasm of ovary
	1832	Malignant neoplasm of fallopian tube
	1833	Malignant neoplasm of broad ligament of uterus
	1834	Malignant neoplasm of parametrium
	1835	Malignant neoplasm of round ligament of uterus
	1838	Malignant neoplasm of other specified sites of uterine adnexa
	1839	Malignant neoplasm of uterine adnexa, unspecified site
	1840	Malignant neoplasm of vagina
	1841	Malignant neoplasm of labia majora

© 2020 Kimmel SD et al. *JAMA Network Open.*

	1842	Malignant neoplasm of labia minora
	1843	Malignant neoplasm of clitoris
	1844	Malignant neoplasm of vulva, unspecified site
	1848	Malignant neoplasm of other specified sites of female genital organs
	1849	Malignant neoplasm of female genital organ, site unspecified
	185	Malignant neoplasm of prostate
	1860	Malignant neoplasm of undescended testis
	1869	Malignant neoplasm of other and unspecified testis
	1871	Malignant neoplasm of prepuce
	1872	Malignant neoplasm of glans penis
	1873	Malignant neoplasm of body of penis
	1874	Malignant neoplasm of penis, part unspecified
	1875	Malignant neoplasm of epididymis
	1876	Malignant neoplasm of spermatic cord
	1877	Malignant neoplasm of scrotum
	1878	Malignant neoplasm of other specified sites of male genital organs
	1879	Malignant neoplasm of male genital organ, site unspecified
	1880	Malignant neoplasm of trigone of urinary bladder
	1881	Malignant neoplasm of dome of urinary bladder
	1882	Malignant neoplasm of lateral wall of urinary bladder
	1883	Malignant neoplasm of anterior wall of urinary bladder
	1884	Malignant neoplasm of posterior wall of urinary bladder
	1885	Malignant neoplasm of bladder neck
	1886	Malignant neoplasm of ureteric orifice
	1887	Malignant neoplasm of urachus
	1888	Malignant neoplasm of other specified sites of bladder
	1889	Malignant neoplasm of bladder, part unspecified
	1890	Malignant neoplasm of kidney, except pelvis
	1891	Malignant neoplasm of renal pelvis
	1892	Malignant neoplasm of ureter
	1893	Malignant neoplasm of urethra
	1894	Malignant neoplasm of paraurethral glands
	1898	Malignant neoplasm of other specified sites of urinary organs
	1899	Malignant neoplasm of urinary organ, site unspecified
	1900	Malignant neoplasm of eyeball, except conjunctiva, cornea, retina, and choroid
	1901	Malignant neoplasm of orbit

© 2020 Kimmel SD et al. *JAMA Network Open.*

	1902	Malignant neoplasm of lacrimal gland
	1903	Malignant neoplasm of conjunctiva
	1904	Malignant neoplasm of cornea
	1905	Malignant neoplasm of retina
	1906	Malignant neoplasm of choroid
	1907	Malignant neoplasm of lacrimal duct
	1908	Malignant neoplasm of other specified sites of eye
	1909	Malignant neoplasm of eye, part unspecified
	1910	Malignant neoplasm of cerebrum, except lobes and ventricles
	1911	Malignant neoplasm of frontal lobe
	1912	Malignant neoplasm of temporal lobe
	1913	Malignant neoplasm of parietal lobe
	1914	Malignant neoplasm of occipital lobe
	1915	Malignant neoplasm of ventricles
	1916	Malignant neoplasm of cerebellum nos
	1917	Malignant neoplasm of brain stem
	1918	Malignant neoplasm of other parts of brain
	1919	Malignant neoplasm of brain, unspecified
	1920	Malignant neoplasm of cranial nerves
	1921	Malignant neoplasm of cerebral meninge
	1922	Malignant neoplasm of spinal cord
	1923	Malignant neoplasm of spinal meninges
	1928	Malignant neoplasm of other specified sites of nervous system
	1929	Malignant neoplasm of nervous system, part unspecified
	193	Malignant neoplasm of thyroid gland
	1940	Malignant neoplasm of adrenal gland
	1941	Malignant neoplasm of parathyroid gland
	1943	Malignant neoplasm of pituitary gland and craniopharyngeal duct
	1944	Malignant neoplasm of pineal gland
	1945	Malignant neoplasm of carotid body
	1946	Malignant neoplasm of aortic body and other paraganglia
	1948	Malignant neoplasm of other endocrine glands and related structures
	1949	Malignant neoplasm of endocrine gland, site unspecified
	1950	Malignant neoplasm of head, face, and neck

© 2020 Kimmel SD et al. *JAMA Network Open.*

	1951	Malignant neoplasm of thorax
	1952	Malignant neoplasm of abdomen
	1953	Malignant neoplasm of pelvis
	1954	Malignant neoplasm of upper limb
	1955	Malignant neoplasm of lower limb
	1958	Malignant neoplasm of other specified sites
	V1000	Personal history of malignant neoplasm of gastrointestinal tract, unspecified
	V1001	Personal history of malignant neoplasm of tongue
	V1002	Personal history of malignant neoplasm of other and unspecified oral cavity and pharynx
	V1003	Personal history of malignant neoplasm of esophagus
	V1004	Personal history of malignant neoplasm of stomach
	V1005	Personal history of malignant neoplasm of large intestine
	V1006	Personal history of malignant neoplasm of rectum, rectosigmoid junction, and anus
	V1007	Personal history of malignant neoplasm of liver
	V1011	Personal history of malignant neoplasm of bronchus and lung
	V1012	Personal history of malignant neoplasm of trachea
	V1020	Personal history of malignant neoplasm of respiratory organ, unspecified
	V1021	Personal history of malignant neoplasm of larynx
	V1022	Personal history of malignant neoplasm of nasal cavities, middle ear, and accessory sinuses
	V1029	Personal history of malignant neoplasm of other respiratory and intrathoracic organs
	V103	Personal history of malignant neoplasm of breast
	V1040	Personal history of malignant neoplasm of female genital organ, unspecified
	V1041	Personal history of malignant neoplasm of cervix uteri
	V1042	Personal history of malignant neoplasm of other parts of uterus
	V1043	Personal history of malignant neoplasm of ovary
	V1044	Personal history of malignant neoplasm of other female genital organs
	V1045	Personal history of malignant neoplasm of male genital organ, unspecified
	V1046	Personal history of malignant neoplasm of prostate
	V1047	Personal history of malignant neoplasm of testis
	V1048	Personal history of malignant neoplasm of epididymis

© 2020 Kimmel SD et al. *JAMA Network Open.*

	V1049	Personal history of malignant neoplasm of other male genital organs
	V1050	Personal history of malignant neoplasm of urinary organ, unspecified
	V1051	Personal history of malignant neoplasm of bladder
	V1052	Personal history of malignant neoplasm of kidney
	V1053	Personal history of malignant neoplasm of renal pelvis
	V1059	Personal history of malignant neoplasm of other urinary organs
	V1060	Personal history of leukemia, unspecified
	V1061	Personal history of lymphoid leukemia
	V1062	Personal history of myeloid leukemia
	V1063	Personal history of monocytic leukemia
	V1069	Personal history of other leukemia
	V1071	Personal history of lymphosarcoma and reticulosarcoma
	V1072	Personal history of hodgkin's disease
	V1079	Personal history of other lymphatic and hematopoietic neoplasms
	V1081	Personal history of malignant neoplasm of bone
	V1082	Personal history of malignant melanoma of skin
	V1083	Personal history of other malignant neoplasm of skin
	V1084	Personal history of malignant neoplasm of eye
	V1085	Personal history of malignant neoplasm of brain
	V1086	Personal history of malignant neoplasm of other parts of nervous system
	V1087	Personal history of malignant neoplasm of thyroid
	V1088	Personal history of malignant neoplasm of other endocrine glands and related structures
	V1089	Personal history of malignant neoplasm of other sites
	V1090	Personal history of unspecified malignant neoplasm
	V1091	Personal history of malignant neuroendocrine tumor

Arthritis	**ICD9**	**ICD9 Description**
	7010	Circumscribed scleroderma
	7101	Systemic sclerosis
	7102	Sicca syndrom
	7103	Dermatomyositis
	7104	Polymyositis
	7105	Eosinophilia myalgia syndrome
	7108	Other specified diffuse diseases of connective tissue

© 2020 Kimmel SD et al. *JAMA Network Open.*

	7108	Unspecified diffuse connective tissue disease
	7140	Rheumatoid arthritis
	7141	Felty's syndrome
	7142	Other rheumatoid arthritis with visceral or systemic involvement
	71430	Polyarticular juvenile rheumatoid arthritis, chronic or unspecified
	71431	Polyarticular juvenile rheumatoid arthritis, acute
	71432	Pauciarticular juvenile rheumatoid arthritis
	71433	Monoarticular juvenile rheumatoid arthritis
	7144	Chronic postrheumatic arthropathy
	71481	Rheumatoid lung
	71489	Other specified inflammatory polyarthropathies
	7149	Unspecified inflammatory polyarthropathy
	7200	Ankylosing spondylitis
	7201	Spinal enthesopathy
	7202	Sacroiliitis, not elsewhere classified
	72081	Inflammatory spondylopathies in diseases classified elsewhere
	72089	Other inflammatory spondylopathies
	7209	Unspecified inflammatory spondylopathy
	725	Polymyalgia rheumatica

Coagulopathy	**ICD9**	**ICD9 Description**
	2860	Congenital factor VIII disorder
	2861	Congenital factor IX disorder
	2862	Congenital factor XI deficiency
	2863	Congenital deficiency of other clotting factors
	2864	Von Willebrand's disease
	28652	Acquired hemophilia
	28653	Antiphospholipid antibody with hemorrhagic disorder
	28659	Other hemorrhagic disorder due to intrinsic circulating anticoagulants, antibodies, or inhibitors
	2866	Defibrination syndrome
	2869	Other and unspecified coagulation defects
	2871	Qualitative platelet defects
	28730	Primary thrombocytopenia,unspecified
	28731	Immune thrombocytopenic purpura
	28732	Evans' syndrome

© 2020 Kimmel SD et al. *JAMA Network Open.*

	28733	Congenital and hereditary thrombocytopenic purpura
	28739	Other primary thrombocytopenia
	28741	Posttransfusion purpura
	38749	Other secondary thrombocytopenia
	2875	Thrombocytopenia, unspecified

Obesity	**ICD9**	**ICD9 Description**
	27800	Obesity, unspecified
	27801	Morbid obesity
	27803	Obesity hypoventilation syndrome

Weight Loss	**ICD9**	**ICD9 Description**
	260	Kwashiorko
	261	Nutritional marasmus
	262	Other severe protein-calorie malnutrition
	2630	Malnutrition of moderate degree
	2631	Malnutrition of mild degree
	2632	Arrested development following protein-calorie malnutrition
	2638	Other protein-calorie malnutrition
	2639	Unspecified protein-calorie malnutrition

Electrolyte Disorders	**ICD9**	**ICD9 Description**
	2760	Hyperosmolality and/or hypernatremia
	2761	Hyposmolality and/or hyponatremia
	2762	Acidosis
	2763	Alkalosis
	2764	Mixed acid-base balance disorder
	27650	Volume depletion, unspecified
	27651	Dehydration
	27652	Hypovolemia
	27661	Transfusion associated circulatory overload
	27669	Other fluid overload
	2767	Hyperpotassemia
	2768	Hypopotassemia
	2769	Electrolyte and fluid disorders not elsewhere classified

Anemia, blood loss	**ICD9**	**ICD9 Description**

© 2020 Kimmel SD et al. *JAMA Network Open.*

	2800	Iron deficiency anemia secondary to blood loss (chronic)

Anemia	**ICD9**	**ICD9 Description**
	2801	Iron deficiency anemia secondary to inadequate dietary iron intake
	2808	Other specified iron deficiency anemias
	2809	Iron deficiency anemia, unspecified
	2810	Pernicious anemia
	2811	Other vitamin B12 deficiency anemia
	2812	Folate-deficiency anemia
	2813	Other specified megaloblastic anemias not elsewhere classified
	2814	Protein-deficiency anemia
	2818	Anemia associated with other specified nutritional deficiency
	2819	Unspecified deficiency anemia
	2859	Anemia, unspecified

Alcohol	**ICD9**	**ICD9 Description**
	2911	Alcohol-induced persisting amnestic disorder
	2912	Alcohol-induced persisting dementia
	2915	Alcohol-induced psychotic disorder with delusions
	29181	Alcohol withdrawal
	29182	Alcohol induced sleep disorders
	29189	Other alcohol-induced mental disorders
	2919	Unspecified alcohol-induced mental disorders
	30390	Other and unspecified alcohol dependence, unspecified
	30391	Other and unspecified alcohol dependence, continuous
	30392	Other and unspecified alcohol dependence, episodic
	30393	Other and unspecified alcohol dependence, in remission
	30500	Alcohol abuse, unspecified
	30501	Alcohol abuse, continuous
	30502	Alcohol abuse, episodic
	30503	Alcohol abuse, in remission
	V113	History of alcoholism

Drug Abuse	**ICD9**	**ICD9 Description**
	2920	Drug withdrawal
	29282	Drug-induced persisting dementia
	29283	Drug-induced persisting amnestic disorder
	29284	Drug-induced mood disorder

© 2020 Kimmel SD et al. *JAMA Network Open.*

	29285	Drug induced sleep disorders
	29289	Other specified drug-induced mental disorders
	2929	Unspecified drug-induced mental disorder
	30400	Opioid type dependence, unspecified
	30401	Opioid type dependence, continuous
	30402	Opioid type dependence, episodic
	30403	Opioid type dependence, in remission
	30550	Opioid abuse, unspecified
	30551	Opioid abuse, continuous
	30552	Opioid abuse, episodic
	30553	Opioid abuse, in remission
	30560	Cocaine abuse, unspecified
	30561	Cocaine abuse, continuous
	30562	Cocaine abuse, episodic
	30563	Cocaine abuse, in remission
	30570	Amphetamine or related acting sympathomimetic abuse, unspecified
	30571	Amphetamine or related acting sympathomimetic abuse, continuous
	30572	Amphetamine or related acting sympathomimetic abuse, episodic
	30573	Amphetamine or related acting sympathomimetic abuse, in remission
	30580	Antidepressant type abuse, unspecified
	30581	Antidepressant type abuse, continuous
	30582	Antidepressant type abuse, episodic
	30583	Antidepressant type abuse, in remission
	30590	Other, mixed, or unspecified drug abuse, unspecified
	30591	Other, mixed, or unspecified drug abuse, continuous
	30592	Other, mixed, or unspecified drug abuse, episodic
	30593	Other, mixed, or unspecified drug abuse, in remission

Depression	**ICD9**	**ICD9 Description**
	3004	Dysthymic disorder
	30112	Chronic depressive personality disorder
	3090	Adjustment disorder with depressed mood
	3091	Prolonged depressive reaction
	311	Depressive disorder, not elsewhere classified

Psychoses	**ICD9**	**ICD9 Description**
	29500	Simple type schizophrenia, unspecified
	29501	Simple type schizophrenia, subchronic
	29502	Simple type schizophrenia, chronic
	29503	Simple type schizophrenia, subchronic with acute exacerbation

© 2020 Kimmel SD et al. *JAMA Network Open.*

	29504	Simple type schizophrenia, chronic with acute exacerbation
	29505	Simple type schizophrenia, in remission
	29550	Latent schizophrenia, unspecified
	29551	Latent schizophrenia, subchronic
	29552	Latent schizophrenia, chronic
	29553	Latent schizophrenia, subchronic with acute exacerbation
	29554	Latent schizophrenia, chronic with acute exacerbation
	29555	Latent schizophrenia, in remission
	29580	Other specified types of schizophrenia, unspecified
	29581	Other specified types of schizophrenia, subchronic
	29582	Other specified types of schizophrenia, chronic
	29583	Other specified types of schizophrenia, subchronic with acute exacerbation
	29584	Other specified types of schizophrenia, chronic with acute exacerbation
	29585	Other specified types of schizophrenia, in remission
	29510	Disorganized type schizophrenia, unspecified
	29511	Disorganized type schizophrenia, subchronic
	29512	Disorganized type schizophrenia, chronic
	29513	Disorganized type schizophrenia, subchronic with acute exacerbation
	29514	Disorganized type schizophrenia, chronic with acute exacerbation
	29515	Disorganized type schizophrenia, in remission
	29520	Catatonic type schizophrenia, unspecified
	29521	Catatonic type schizophrenia, subchronic
	29522	Catatonic type schizophrenia, chronic
	29523	Catatonic type schizophrenia, subchronic with acute exacerbation
	29524	Catatonic type schizophrenia, chronic with acute exacerbation
	29525	Catatonic type schizophrenia, in remission
	29530	Paranoid type schizophrenia, unspecified
	29531	Paranoid type schizophrenia, subchronic
	29532	Paranoid type schizophrenia, chronic
	29533	Paranoid type schizophrenia, subchronic with acute exacerbation
	29534	Paranoid type schizophrenia, chronic with acute exacerbation
	29535	Paranoid type schizophrenia, in remission
	29540	Schizophreniform disorder, unspecified
	29541	Schizophreniform disorder, subchronic
	29542	Schizophreniform disorder, chronic
	29543	Schizophreniform disorder, subchronic with acute exacerbation
	29544	Schizophreniform disorder, chronic with acute exacerbation
	29545	Schizophreniform disorder, in remission

© 2020 Kimmel SD et al. *JAMA Network Open.*

	29560	Schizophrenic disorders, residual type, unspecified
	29561	Schizophrenic disorders, residual type, subchronic
	29562	Schizophrenic disorders, residual type, chronic
	29563	Schizophrenic disorders, residual type, subchronic with acute exacerbation
	29564	Schizophrenic disorders, residual type, chronic with acute exacerbation
	29565	Schizophrenic disorders, residual type, in remission
	29670	Schizoaffective disorder, unspecified
	29671	Schizoaffective disorder, subchronic
	29672	Schizoaffective disorder, chronic
	29673	Schizoaffective disorder, subchronic with acute exacerbation
	29674	Schizoaffective disorder, chronic with acute exacerbation
	29675	Schizoaffective disorder, in remission
	29590	Unspecified schizophrenia, unspecified
	29591	Unspecified schizophrenia, subchronic
	29592	Unspecified schizophrenia, chronic
	29593	Unspecified schizophrenia, subchronic with acute exacerbation
	29594	Unspecified schizophrenia, chronic with acute exacerbation
	29595	Unspecified schizophrenia, in remission
	29600	Bipolar I disorder, single manic episode, unspecified
	29610	Manic affective disorder, recurrent episode, unspecified
	29601	Bipolar I disorder, single manic episode, mild
	29611	Manic affective disorder, recurrent episode, mild
	29602	Bipolar I disorder, single manic episode, moderate
	29613	Manic affective disorder, recurrent episode, severe, without mention of psychotic behavior
	29603	Bipolar I disorder, single manic episode, severe, without mention of psychotic behavior
	29612	Manic affective disorder, recurrent episode, moderate
	29604	Bipolar I disorder, single manic episode, severe, specified as with psychotic behavior
	29614	Manic affective disorder, recurrent episode, severe, specified as with psychotic behavior
	29605	Bipolar I disorder, single manic episode, in partial or unspecified remission
	29615	Manic affective disorder, recurrent episode, in partial or unspecified remission
	29606	Bipolar I disorder, single manic episode, in full remission
	29616	Manic affective disorder, recurrent episode, in full remission

© 2020 Kimmel SD et al. *JAMA Network Open.*

	29620	Major depressive affective disorder, single episode, unspecified
	29621	Major depressive affective disorder, single episode, mild
	29622	Major depressive affective disorder, single episode, moderate
	29623	Major depressive affective disorder, single episode, severe, without mention of psychotic behavior
	29624	Major depressive affective disorder, single episode, severe, specified as with psychotic behavior
	29625	Major depressive affective disorder, single episode, in partial or unspecified remission
	29626	Major depressive affective disorder, single episode, in full remission
	29630	Major depressive affective disorder, recurrent episode, unspecified
	29631	Major depressive affective disorder, recurrent episode, mild
	29632	Major depressive affective disorder, recurrent episode, moderate
	29633	Major depressive affective disorder, recurrent episode, severe, without mention of psychotic behavior
	29634	Major depressive affective disorder, recurrent episode, severe, specified as with psychotic behavior
	29635	Major depressive affective disorder, recurrent episode, in partial or unspecified remission
	29636	Major depressive affective disorder, recurrent episode, in full remission
	29640	Bipolar I disorder, most recent episode (or current) manic, unspecified
	29641	Bipolar I disorder, most recent episode (or current) manic, mild
	29642	Bipolar I disorder, most recent episode (or current) manic, moderate
	29643	Bipolar I disorder, most recent episode (or current) manic, severe, without mention of psychotic behavior
	29644	Bipolar I disorder, most recent episode (or current) manic, severe, specified as with psychotic behavior
	29645	Bipolar I disorder, most recent episode (or current) manic, in partial or unspecified remission
	29646	Bipolar I disorder, most recent episode (or current) manic, in full remission
	29650	Bipolar I disorder, most recent episode (or current) depressed, unspecified

© 2020 Kimmel SD et al. *JAMA Network Open.*

	29651	Bipolar I disorder, most recent episode (or current) depressed, mild
	29652	Bipolar I disorder, most recent episode (or current) depressed, moderate
	29653	Bipolar I disorder, most recent episode (or current) depressed, severe, without mention of psychotic behavior
	29654	Bipolar I disorder, most recent episode (or current) depressed, severe, specified as with psychotic behavior
	29655	Bipolar I disorder, most recent episode (or current) depressed, in partial or unspecified remission
	29656	Bipolar I disorder, most recent episode (or current) depressed, in full remission
	29660	Bipolar I disorder, most recent episode (or current) mixed, unspecified
	29660	Bipolar I disorder, most recent episode (or current) mixed, mild
	29660	Bipolar I disorder, most recent episode (or current) mixed, moderate
	29660	Bipolar I disorder, most recent episode (or current) mixed, severe, without mention of psychotic behavior
	29660	Bipolar I disorder, most recent episode (or current) mixed, severe, specified as with psychotic behavior
	29660	Bipolar I disorder, most recent episode (or current) mixed, in partial or unspecified remission
	29660	Bipolar I disorder, most recent episode (or current) mixed, in full remission
	2967	Bipolar I disorder, most recent episode (or current) unspecified
	29680	Bipolar disorder, unspecified
	29681	Atypical manic disorder
	29682	Atypical depressive disorder
	29689	Other bipolar disorders
	29690	Unspecified episodic mood disorder
	29699	Other specified episodic mood disorder
	2970	Paranoid state, simple
	2971	Delusional disorder
	2972	Paraphrenia
	2978	Other specified paranoid states
	2973	Shared psychotic disorder
	2979	Unspecified paranoid state
	2983	Acute paranoid reaction
	2984	Psychogenic paranoid psychosis
	2988	Other and unspecified reactive psychosis
	2980	Depressive type psychosis
	2981	Excitative type psychosis

© 2020 Kimmel SD et al. *JAMA Network Open.*

	2982	Reactive confusion
	2989	Unspecified psychosis
	29910	Childhood disintegrative disorder, current or active state
	29911	Childhood disintegrative disorder, residual state

© 2020 Kimmel SD et al. *JAMA Network Open.*

e Figure 3: Cohort Construction, 2011-2015

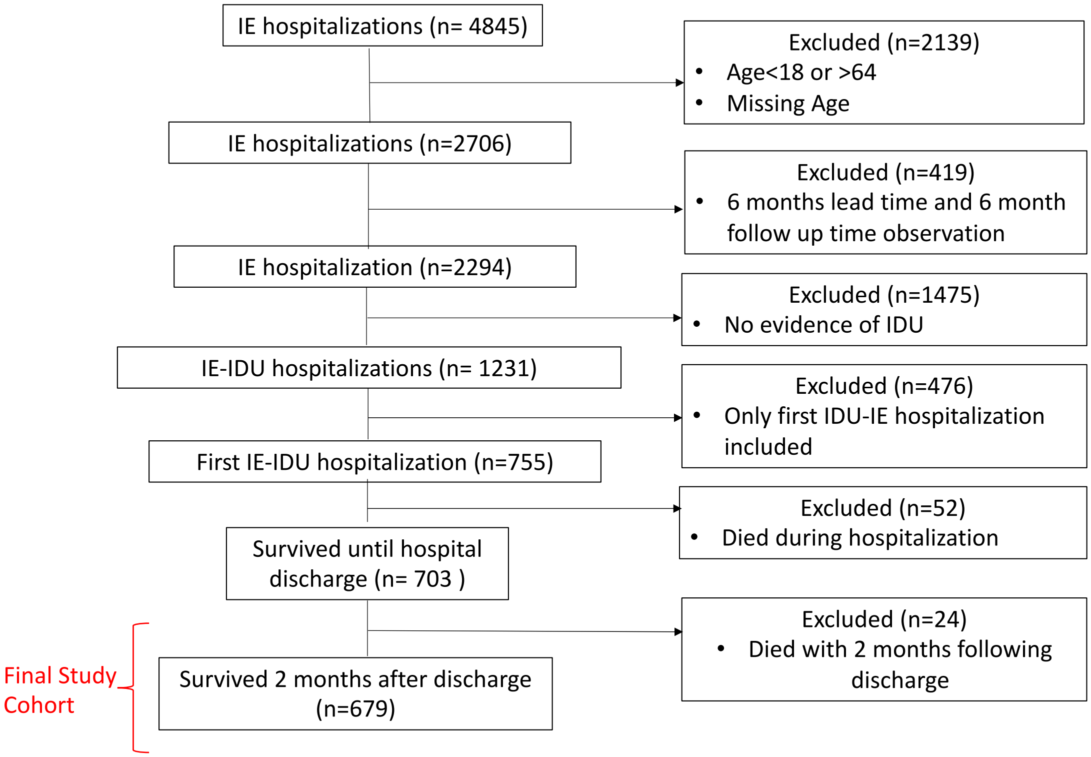

© 2020 Kimmel SD et al. *JAMA Network Open.*

e Table 4: Sensitivity Analyses: Adjusted* Cox Proportional Hazard Models for association of MOUD with All-Cause Mortality, July 2011 to June 2015

			Model 1	Model 2	Model 3
	Sample size	Deaths	MOUD within 3 Months Following IDU-IE (Cox Proportional Hazard)	Monthly MOUD Receipt following IDU-IE (Time Varying Cox Proportional Hazard)	Monthly, including month of discontinuation, following IDU-IE (Time Varying Cox Proportional Hazard)
Main model	679	61	1.29 (0.61-2.72)	0.30 (0.10-0.89)	0.39 (0.15-1.00)
Hepatitis C removed from inclusion criteria	621	52	1.25 (0.60-2.64)	0.30 (0.10-0.87)	0.38 (0.15-0.98)
Upper age limit removed from inclusion criteria	712	70	1.15 (0.55-2.40)	0.28 (0.10-0.84)	0.37 (0.15-0.93)
Minimum survival requirement, month of discharge	693	75	0.74 (0.36-1.52)	0.35 (0.14-0.89)	0.27 (0.09-0.80)

*Models adjusted for MOUD 3 months prior to admission, age, sex, Elixhauser score, major mental illness, homelessness.

© 2020 Kimmel SD et al. *JAMA Network Open.*
